# Taxonomic revision of the olingos (*Bassaricyon*), with description of a new species, the Olinguito

**DOI:** 10.3897/zookeys.324.5827

**Published:** 2013-08-15

**Authors:** Kristofer M. Helgen, C. Miguel Pinto, Roland Kays, Lauren E. Helgen, Mirian T. N. Tsuchiya, Aleta Quinn, Don E. Wilson, Jesús E. Maldonado

**Affiliations:** 1 Division of Mammals, National Museum of Natural History, NHB 390, MRC 108, Smithsonian Institution, P.O. Box 37012, Washington, DC 20013-7012, USA; 2 Centro de Investigación en Enfermedades Infecciosas, Escuela de Ciencias Biológicas, Pontificia Universidad Católica del Ecuador, Av. 12 de Octubre y Roca, Quito, Ecuador; 3 Department of Mammalogy, and Sackler Institute for Comparative Genomics, American Museum of Natural History, Central Park West at 79th Street, New York, NY 10024, USA; 4 The Graduate Center, City University of New York, 365 Fifth Ave., New York, NY, 10016 USA; 5 Department of Biological Sciences and the Museum, Texas Tech University, Lubbock, Texas 79409-3131, USA; 6 North Carolina Museum of Natural Sciences, 11 West Jones Street, Raleigh, NC, 27601, USA; 7 Fisheries, Wildlife & Conservation Program, North Carolina State University, Raleigh, NC, 27695, USA; 8 Smithsonian Tropical Research Institute, Balboa Ancón, Republic of Panamá; 9 Department of Environmental Science & Policy, George Mason University, Fairfax, VA, 22030 USA; 10 Center for Conservation and Evolutionary Genetics, Smithsonian Conservation Biology Institute, National Zoological Park, Washington, DC 20008, USA; 11 Department of History and Philosophy of Science, University of Pittsburgh, Pittsburgh, PA, 15260, USA

**Keywords:** Andes, *Bassaricyon*, biogeography, Neotropics, new species, olingo, Olinguito

## Abstract

We present the first comprehensive taxonomic revision and review the biology of the olingos, the endemic Neotropical procyonid genus *Bassaricyon*, based on most specimens available in museums, and with data derived from anatomy, morphometrics, mitochondrial and nuclear DNA, field observations, and geographic range modeling. Species of *Bassaricyon* are primarily forest-living, arboreal, nocturnal, frugivorous, and solitary, and have one young at a time. We demonstrate that four olingo species can be recognized, including a Central American species (*Bassaricyon gabbii*), lowland species with eastern, cis-Andean (*Bassaricyon alleni*) and western, trans-Andean (*Bassaricyon medius*) distributions, and a species endemic to cloud forests in the Andes. The oldest evolutionary divergence in the genus is between this last species, endemic to the Andes of Colombia and Ecuador, and all other species, which occur in lower elevation habitats. Surprisingly, this Andean endemic species, which we call the Olinguito, has never been previously described; it represents a new species in the order Carnivora and is the smallest living member of the family Procyonidae. We report on the biology of this new species based on information from museum specimens, niche modeling, and fieldwork in western Ecuador, and describe four Olinguito subspecies based on morphological distinctions across different regions of the Northern Andes.

## Introduction

“New Carnivores of any sort are always few and far between…”

Oldfield Thomas (1894:524)

Olingos (genus *Bassaricyon* J.A. Allen, 1876) are small to medium-sized (0.7 to 2 kg) arboreal procyonids found in the forests of Central America and northern South America. No comprehensive systematic revision of the genus has ever been undertaken, such that species boundaries in *Bassaricyon* are entirely unclear, and probably more poorly resolved than in any other extant carnivoran genus. There are various reasons for limited knowledge of *Bassaricyon*. For such a widespread genus of Carnivora, olingos were discovered surprisingly late (first described from Central America in 1876 and from South America in 1880; Allen 1876, Thomas 1880); they were long known by few specimens in museum collections; they are often overlooked in the field because they are regularly confused with another better known procyonid, the kinkajou, *Potos flavus* (Schreber, 1774) (e.g., Thomas 1880, Manville 1956, Ford and Hoffmann 1988, Sampaio et al. 2011); and they are often largely or entirely omitted from both authoritative and popular references on Neotropical wildlife and natural history (e.g., Janzen 1983, Henderson 2002, Lord 2007). In the absence of a detailed systematic review, five species of *Bassaricyon* are tentatively recognized in most recent taxonomic references, including three species in Central America (*Bassaricyon gabbii* Allen, 1876; *Bassaricyon lasius* Harris, 1932; *Bassaricyon pauli* Enders, 1936) and three species in South America (with *Bassaricyon gabbii* recognized as occurring west of the Andes, and *Bassaricyon alleni* Thomas, 1880 and *Bassaricyon beddardi* Pocock, 1921a east of the Andes), but most authors have explicitly identified a longstanding need for a detailed taxonomic overview to clarify species diversity and distributions in this genus (Cabrera 1958, Decker and Wozencraft 1991, Eisenberg 1989, Eisenberg and Redford 1999, Eizirik 2012, Emmons 1990, 1997, Ewer 1973, Glatston 1994, González-Maya et al. 2011, Hall 1981, Hall and Kelson 1959, Helgen et al. 2008c, Hunter 2011, Kays 2009, Kays and Russell 2001, Nowak 1999, Poglayen-Neuwall and Poglayen-Neuwall 1965, Prange and Prange 2009, Reid 1997, 2009, Reid and Helgen 2008a, 2008b, 2008c, Russell 1984, Samudio et al. 2008, Stains 1967, Wozencraft 1989, 1993, 2005, Zeveloff 2002).

Here we review the taxonomic standing of all named forms of *Bassaricyon* based on morphological, morphometric, and molecular comparisons of voucher specimens in museums; we clarify the distribution and conservation status of each valid taxon; and, as far as possible, we enable information from published literature on olingo anatomy (e.g., Beddard 1900, Mivart 1885, 1886, Pocock 1921a, 1921b, Segall 1943, Stains 1973, Story 1951), ecology and behavior (e.g., Aquino and Encarnación 1986, Emmons 1990, 1991, Glanz 1990, Goldman 1920, Hunter and Caro 2008, Janson and Emmons 1990, Kays 2000, Loyola et al. 2008, Patton et al. 1982, Peres 2001, Poglayen-Neuwall 1966, 1973, 1976, 1989, Poglayen-Neuwall and Poglayen-Neuwall 1965, Prange and Prange 2009, Reid 1997, Rodríguez and Amanzo 2001, Wainwright 2002), and parasites and disease (e.g., Grimaldi and Tesh 1993, Hendricks 1977, Herrer and Christensen 1975, Jewell et al. 1972, Magalhães-Pinto et al. 2009) to be associated with particular olingo taxa now recognized as valid.

All previously described olingo taxaoccur in lower to middle-elevation tropical or subtropical forests (≤ 2000 meters in elevation). Remarkably, our morphological, morphometric, molecular, and field studies document the existence of an undescribed species in the genus, endemic to higher-elevation cloud forests (1500 to 2750 meters) in the Western and Central Andes of Colombia and Ecuador, which we describe here as a new species. (This species has been discussed preliminarily, in advance of its formal description, by Kays [2009] and Hunter [2011].) This species, upon which we bestow the common name of Olinguito (oh-ling-GHEE’-toh), is the sister taxon to a lineage comprising all previously described species of *Bassaricyon*; is the smallest living procyonid; and is the first new species of American carnivore described since the discovery of the Colombian weasel (*Mustela felipei*) in similar habitats in the same region of the Andes more than three decades ago (Izor and de la Torre 1978). We discuss what is known to date of the biology of this remarkable new procyonid, the Olinguito.

## Materials and methods

### Museum specimens and comparisons

We examined all *Bassaricyon* specimens in the collections of the American Museum of Natural History, New York, USA (AMNH); Academy of Natural Sciences, Philadelphia, USA (ANSP); Natural History Museum, London, UK (BMNH); Museo de Zoología, Universidad Politecnica, Quito, Ecuador (EPN); Field Museum of Natural History, Chicago, USA (FMNH); Biodiversity Institute, University of Kansas, Lawrence, USA (KU); Los Angeles County Natural History Museum, Los Angeles, USA (LACM); Museum of Comparative Zoology, Harvard University, Cambridge, USA (MCZ); Museo Ecuatoriano de Ciencias Naturales, Quito, Ecuador (MECN); Museum of Vertebrate Zoology, University of California, Berkeley, USA (MVZ); Naturhistoriska Riksmuseet, Stockholm, Sweden (NMS); Museo de Zoología, Pontificia Universidad Católica del Ecuador, Quito, Ecuador (QCAZ); Royal Ontario Museum, Toronto, Canada (ROM); Biodiversity Research and Teaching Collections, Texas A&M University, College Station, USA (TCWC); Museum of Zoology, University of Michigan, Ann Arbor, USA (UMMZ); National Museum of Natural History, Smithsonian Institution, Washington, D.C., USA (USNM); Peabody Museum of Natural History, Yale University, New Haven, USA (YPM); and Museum für Naturkunde, Humboldt Universität, Berlin, Germany (ZMB). These holdings include all type specimens in the genus and represent the great majority (well over 95%) of olingo specimens in world museums. We also had access to published information on a few additional specimens in museum collections in Colombia and Bolivia (Saavedra-Rodríguez and Velandia-Perilla 2011, Anderson 1997). Tissue samples are stored in the frozen tissue collections of the MVZ, ROM, USNM (including specimens to be accessioned at QCAZ), the New York State Museum, Albany, New York, USA (NYSM), and the Museum of Texas Tech University, Lubbock, Texas, USA (TTU) (Table 1).

**Table 1.** List of samples (and associated information) used in phylogenetic analysis. Boldfaced entries represent samples newly sequenced in this study.

SPECIES	Identifier in Figure 1	Specific locality	Source (catalog reference)	Genbank Accession Numbers	
				Cytochrome *b*	*CHRNA1*
***Bassaricyon medius orinomus***	**Panama**	**Limbo plot**	**NYSM ZT105**	**EF107703**	**KC773757**
***Bassaricyon medius orinomus***	**Panama**	**Rio Juan Grande**	**NYSM ZT106**	**EF107704**	**KC773758**
*Bassaricyon medius orinomus*	Panama	Limbo plot	Koepfli et al. (2007)	DQ660300	DQ660210
***Bassaricyon medius medius***	**Ecuador**	**Las Pampas**	**QCAZ 8659; tk149097**	**EF107706**	**KC773759**
***Bassaricyon medius medius***	**Ecuador**	**Las Pampas**	**QCAZ 8658; tk149094**	**EF107707**	**KC773760**
***Bassaricyon alleni***	**Guyana**	**Iwokrama**	**ROM 107380**	**EF107710**	**KC773763**
*Bassaricyon alleni*	Peru	Rio Cenapa	MVZ 155219; Koepfli et al. (2007)	DQ660299	DQ660209
***Bassaricyon gabbii***	**Costa Rica**	**Monteverde**	**KU 165554**	**JX948744**	**---**
***Bassaricyon neblina neblina***	**Ecuador**	**La Cantera**	**QCAZ 8662; tk149108**	**EF107708**	**KC773761**
***Bassaricyon neblina neblina***	**Ecuador**	**Otonga Reserve**	**QCAZ 8661; tk149001**	**EF107709**	**KC773762**
*Bassaricyon neblina osborni*	Colombia	Vicinity of Cali	Genbank	X94931	DQ533950
*Potos flavus*	*Potos flavus*	Costa Rica	Koepfli et al. (2007)	DQ660304	DQ660214
*Procyon cancrivorus*	*Procyon cancrivorus*	Paraguay	Koepfli et al. (2007)	DQ660305	DQ660215
*Procyon lotor*	*Procyon lotor*	Montana, USA	Koepfli et al. (2007)	DQ660306	AF498152
*Bassariscus astutus*	*Bassariscus astutus*	Arizona, USA	Koepfli et al. (2007)	AF498159	AF498151
*Bassariscus sumichrasti*	*Bassariscus sumichrasti*	Mexico	Koepfli et al. (2007)	DQ660301	DQ660211
*Nasua nasua*	*Nasua nasua*	Bolivia	Koepfli et al. (2007)	DQ660303	DQ660213
*Nasua narica*	*Nasua narica*	Panama	Koepfli et al. (2007)	DQ660302	DQ660212
*Enhydra lutris*	Mustelidae	Attu Island, Alaska, USA	Koepfli et al. (2007)	AF057120	AF498131
*Eira barbara*	Mustelidae	Bolivia	Koepfli et al. (2007)	AF498154	AF498144
*Taxidea taxus*	Mustelidae	New Mexico, USA	Koepfli et al. (2007)	AF057132	AF498148
*Neovison vison*	Mustelidae	Texas, USA	Koepfli et al. (2007)	AF057129	AF498140
*Martes americana*	Mustelidae	Rocky Mtn Research Station, USA	Koepfli et al. (2007)	AF057130.1	AF498141
*Lontra longicaudis*	Mustelidae	Kagka, Peru	Koepfli et al. (2007)	AF057123.1	AF498134
*Ictonyx libyca*	Mustelidae	Brookfield Zoo	Genbank	EF987739.1	EF987699
*Meles meles*	Mustelidae	No voucher infromation	Koepfli et al. (2007)	AM711900.1	AF498147
*Mephitis mephitis*	Mephitidae	San Diego Zoo	Eizirik et al. (2010), Yu et al. (2011)	HM106332.1	GU931029.1
*Spilogale putorius*	Mephitidae		Arnason et al. (2007), Eizirik et al. (2010)	NC_010497.1	GU931030.1
*Ailurus fulgens*	Ailuridae		Arnason et al. (2007), Eizirik et al. (2010)	AM711897.1	GU931037.1
*Arctocephalus australis*	Otariidae		Davis et al. (2004), Fulton and Strobeck (2006)	AY377329.1	DQ205738.1
*Odobenus rosmarus*	Odobenidae		Bardeleben et al. (2005), Fulton and Strobeck (2010)	GU174611.1	DQ093076.1
*Phoca fasciata*	Phocidae		Fulton and Strobeck (2010)	GU167294.1	GU167764.1
*Mirounga angustirostris*	Phocidae		Bardeleben et al. (2005), Peng et al. (2007)	AY377325.1	DQ093075.1
*Canis lupus*	*Canis lupus*		Delisle and Strobeck (2005), Fulton and Strobeck (2006)	AY598499	DQ205757
*Nyctereutes procyonoides*	other Canidae		Eizirik et al. (2010), Chen and Zhang (2012)	GU256221	GU931027.1
*Urocyon cinereoargenteus*	other Canidae		Eizirik et al. (2010), Naidu et al. (2012)	JF489121.1	GU931028.1
*Ailuropoda melanoleuca*	Ursidae		Bardeleben et al. (2005), Peng et al. (2007)	NC_009492	DQ093074.1
*Ursus americanus*	Ursidae		Delisle and Strobeck (2002), Fulton and Strobeck (2006)	NC_003426.1	DQ205726.1

Values from external measurements of 95 specimens are presented to provide an appreciation of general body size and lengths and proportions of appendages. Values (in mm) for total length and length of tail are those recorded by collectors on labels attached to skins; subtracting length of tail (abbreviated TV) from total length produced a value for length of head and body (HB). Values for length of hind foot (HF), which includes claws, were either obtained from skin labels or from our measurements of dry study skins; those for length of external ear (E), or pinna, come from collector’s measurements recorded on skin labels or in field journals (we assume, but are not certain for all specimens, that ear-length measurements represent the greatest length from the notch to the distal margin of the pinna).

Morphological terminology follows Evans (1993) and Ahrens (2012). Craniodental variables were measured by the first author with digital calipers to the nearest 0.1 mm. Single-tooth measurements are measured on the crown. All measurements of length are in millimeters, and measurements of mass are given in grams. Only fully adult, wild-collected specimens that are sufficiently intact were included in our morphometric analyses. A total of 115 specimens were included (51 male, 64 female). The classification of ‘‘adult’’ was applied generally only to skulls in which the full dentition is completely erupted, and in which the basilar (basioccipital-basisphenoid) suture (synchondrosis) in particular is obliterated via ossification. Variables measured include maximum crown widths (W) of premolars (p1, p2, p3, p4, P2, P3, P4, with lower case designating lower teeth and uppercase designating upper teeth) and molars (m1, m2, M1, and M2); maximum crown lengths (L) of the larger premolars and molars (P4, M1, M2, m1, and m2); condylobasal length (CBL), zygomatic width (ZYG), breadth of braincase (BBC), external width across the canines (CC), and length of the maxillary toothrow, C-M2 (MTR), all as defined by Kennedy and Lindsay (1984); and four posterior skull measurements: greatest width across the postdental palatal shelf (WPP), length of the postdental palate behind an imaginary line delineated by the back of the second molars (LPP), anteroposterior length of the auditory bullae including the eustachian tube (LAB), and the dorsoventral diameter inside the external auditory meatus (EAM). Unless explicitly noted, all reported metrics (and resulting statistical and multivariate comparisons) refer only to fully mature (adult) specimens, as judged by direct examination of skulls. Because some olingo taxa demonstrate significant sexual dimorphism in cranial measurements, patterns of morphometric variation in males and females were compiled and analyzed separately. Principal Component Analysis (PCA) and Discriminant Function Analyses (DFA) were undertaken using a combination of cranial and dental measurements indicated in tables and in the text, selected to sample craniodental size and shape, and to maximize sample size. All measurement values were transformed to natural logarithms prior to multivariate analysis. Principal components were extracted from the covariance matrix. The software program Statistica 8.0 (Statsoft Inc., Tulsa, Oklahoma, USA) was used for all multivariate analyses.

The taxa and sequences included in our analysis are listed in Table 1. Our choice of taxa outside of *Bassaricyon* was guided by the findings of Koepfli et al. (2007), Fulton and Strobeck (2007), and Eizirik et al. (2010). These studies provide strong statistical support for relationships and divergence dates within Procyonidae and Carnivora based on >6,000 bases of DNA and fossil evidence. We chose one mitochondrial marker and one nuclear marker used in these and many other mammalian studies, in order to capture the evolutionary histories of these distinct genetic systems. Although deeper relationships within the order Carnivora cannot be resolved solely by using these two genes, we are confident that they provide the appropriate level of support to resolve species-level relationships within this group of procyonids (Koepfli et al. 2007). As our specific goal was to estimate the timing of divergence within *Bassaricyon*, and our reduced dataset did not provide enough support to resolve deeper nodes in Caniformia, we decided to use highly supported divergence date estimates from Koepfli et al. (2007) and Eizirik et al. (2010) as priors in our analysis.

### DNA extraction

Tissues from fresh and frozen specimens were processed using a Qiagen DNeasy kit (QIAGEN, Valencia, CA, USA) to obtain genomic DNA. The sample from the skull of KU 165554, a museum specimen of *Bassaricyon gabbii*, was taken from the turbinate bones and extracted following the method of Wisely et al. (2004). Including this turbinate sample of *Bassaricyon gabbii*, we successfully extracted DNA from eight individuals of *Bassaricyon* (four *Bassaricyon medius*, one *Bassaricyon alleni*, and two *Bassaricyon neblina* sp. n.). All pre-PCR protocols were conducted in an isolated ancient DNA laboratory facility located in a separate building from the one containing the primary DNA laboratory.

### DNA Sequencing

**Mitochondrial gene, cytochrome *b*:** For cytochrome *b* (1140 bp), polymerase chain reaction (PCR) and sequencing reactions were carried out with primers LGL 765 and LGL 766 from Bickham et al. (2004) and using a thermal cycler (MJ Research, Waltham, MA, USA) under the following conditions, repeated for 35 cycles: denaturation at 92°C for 1 min, annealing at 50°C for 1 min, extension at 72°C for 1 min. The PCR reagents in a 25 μL reaction were 0.2 μL AmpliTaq (5 units μL-1, Applied Biosystems, Foster City, CA, USA), 1μL per primer (10 μM), 2.5 μL dNTP (2 μM), 2 μL MgCl2 (25 mM), 2.5 μL AmpliTaq Buffer (Applied Biosystems), 2μL BSA (0.01 mg/μL), 1 μL gDNA and 12.8 μL sterile water. To amplify DNA from turbinate samples, PCR and sequencing were carried out with internal primers designed for this study using sequences generated from the tissue samples; the reverse primer, H151949Pro (5’ CTCCTCAAAAGGATATTTGYCCTCA 3’), located at 14611 – 14636 on the *Nasua nasua* mitochondrial genome, was used with LGL 765. A new forward primer, BAS420F (5’ TCAGACAAAATCCCCTTCCA 3’), position 14825 - 14845 on the *Nasua nasua* mitochondrial genome, was used with LGL 766. The thermal cycle regime was modified to 50 cycles; reagents were as above.

**Nuclear intron, Cholinergic Receptor Nicotinic Alpha Polypeptide 1 precursor (*CHRNA1*):** For *CHRNA1* (347 bp), we used the primers described by Lyons et al. (1997) and the thermocycling conditions consisted of an initial denaturation (95°C for 10 min), followed by 30 cycles of denaturation at 95°C for 30 s, annealing at 54°C for 30 s and extension for 72°C for 45 s, and final extension of 72°C for 5 min. Reagent volumes were the same as for cytochrome *b* amplification (above), except 2μL of gDNA was added for *CHRNA1* amplification, decreasing sterile water volume to 10.8μL. We were unable to sequence the nuclear intron from the turbinate bone sample.

Each PCR was conducted with negative and positive controls to minimize risk of spurious results from contamination or failure of the reaction. A 2μL sample of the PCR product was stained with ethidium bromide and run on an agarose gel with a 1 kb ladder. The gel was placed under UV light to visualize the PCR products. Polymerase chain reaction products were amplified for sequencing using a 10 μL reaction mixture of 2 μL of PCR product, 0.8 μL of primer (10 μM), 1.5 μL Big Dye 5 x Buffer (Applied Biosystems), 1 μL Big Dye version 3 (Applied Biosystems), and 4.7 μL sterile water. The reaction was run using a thermal cycler (MJ Research) with denaturation at 96°C for 10 s, annealing at 50°C for 10 s and extension at 60°C for 4 min: this was repeated for 25 cycles. The product was cleaned using sephadex filtration method and sequences for both strands were run on a 50 cm array using the ABI PRISM 3100 Genetic Analyzer (Applied Biosystems).

### Molecular analysis

Sequences were aligned and edited in Sequencher version 4.1.2 using the implemented Clustal algorithm and the default gap penalty parameters (Gene Codes Corporation, Ann Arbor, MI, USA, http://www.genecodes.com).

For *Bassaricyon*, we included all newly sequenced and previously available sequences for cytochrome *b* and *CHRNA1* (for cytochrome *b* this included five individuals of *Bassaricyon medius*, two *Bassaricyon alleni*, one *Bassaricyon gabbii* and three *Bassaricyon neblina* sp. n.; for *CHRNA1* this included five individuals of *Bassaricyon medius*, two *Bassaricyon alleni*, and three *Bassaricyon neblina* sp. n.) (Table 1).

Maximum Parsimony, Maximum Likelihood and Bayesian analyses were performed for each gene and a concatenation of the two genes to check for any incongruence in structure and support of the *Bassaricyon* clade. All Bayesian and Maximum Likelihood phylogenetic inferences were carried out using the Cipres Portal (Miller et al. 2009). Indels were treated as missing data or non-informative data for all of the analyses as in previous molecular phylogenetic studies of procyonids (Koepfli et al. 2007; Eizirik et al. 2010).

Pairwise distances for cytochrome *b* were generated using the Kimura 2-parameter model using MEGA4 (Tamura et al. 2007).

The branch and bound search method implemented in the software package TNT (Goloboff et al. 2008) was used for the maximum parsimony analyses. Parsimony bootstrap support was generated using the heuristic search method with 100 random stepwise additions for 1000 replicates.

Maximum Likelihood analysis was conducted using the software package GARLI 0.96b (Zwickl 2006). The genetic-like algorithm was used to simultaneously find the topology, branch lengths and substitution model parameters with the greatest log-likelihood (lnL) for each dataset. Bootstrap support was generated with 1000 replicates and two independent searches per replicate.

jModeltest version 0.1.1 (Posada 2008) was used to find the best model of sequence evolution. We chose to partition the cytochrome *b* data in order to minimize the number of parameters and to account for differences in base composition and substitution rates among the different codon positions (Corse et al. 2013). The software PartitionFinder (Lanfear et al. 2012) was used to determine the best partitioning scheme, and for the cytochrome *b*, the scheme with 1^st^, 2^nd^, and 3^rd^ codon positions partitioned separately was selected. The best fit model under the Bayesian information criterion (BIC) for cytochrome *b* for the first and second codon position partitions was HKY + G + I (Hasegawa et al. 1985), and for the third codon position, the best model under BIC was TrN + I + G (Tamura and Nei 1993). The model chosen for *CHRNA1* was K80 + G (Kimura 1980). The parameters were then applied in MrBayes version 3.1p (Huelsenbeck and Ronquist 2001). The model parameters were set to nst = 2 using a gamma distribution for *CHRNA1*, nst = 2 and the rate parameter invariant with a gamma distribution for the cytochrome *b* 1st and 2nd codon partitions, and nst = 6 with a gamma distribution and rate parameter invariant for cytochrome *b* 3rd codon partition. Since this version of MrBayes did not include the specific model selected for the cytochrome *b* 3rd codon position partition, we opted for using a more complex model (nst = 6) following the results of Huelsenbeck and Rannala (2004). The Bayesian analysis was run using 5,000,000 generations along four chains with 2 replicates at a temperature of 0.05. The convergence between the two replicates run in MrBayes was assessed by the average standard deviation of split frequencies (ASDSF) between runs. After 5,000,000 generations, the ASDSF was 0.003. Sample frequency was set to 1000 with a burn-in of 1,250.

Molecular divergence estimates were generated in BEAST (Drummond and Rambaut 2007). The following calibration nodes were included based on Eizirik et al. (2010): *Nasua* – *Bassaricyon* truncated normal mean 7.2 million years ago (mya) (± 1.7 s.d.); -*Potos* truncated normal mean 16.2 mya (± 2.5 s.d.); Procyonidae normal mean 20.7 mya (± 4.0 s.d.); Procyonidae-Mustelidae-Ailuridae-Mephitidae normal mean 30 mya (± 7.0 s.d.); *Phoca*-*Mirounga* normal mean 20 mya (± 6 s.d.); Caniformia normal mean 48 mya (± 6.5 s.d.). The molecular clock was estimated using the uncorrelated lognormal setting, operators were left to the default setting, and trees were searched using the Yule process. The substitution and clock models were left unlinked, partition tree model was linked, and the models for the two gene partitions were: cytochrome *b* (1), (2), and (3) => TN93 + I + G (all parameters unlinked) and *CHRNA1* K80 + G (HKY + G). In order to evaluate the effects of the priors on the divergence time estimates, we carried out a run using an empty alignment but with the same settings and compared it to our results,with the outcome indicating that the priors are not having an especially strong effect on the estimated divergence times (Drummond et al. 2006).

To infer geographical range evolution of procyonids we used the Maximum Likelihood model of dispersal-extinction-cladogenesis (DEC) implemented in Lagrange v. 20130526 (Ree and Smith 2008). The BEAST chronogram tree was trimmed to keep one representative per procyonid species, and two additional lineages, one representing Mustelidae and one representing Mephitidae. Six general geographic areas were used to characterize the distribution ranges: Eurasia, North America, Central America, Chocó, Andes, and Amazonia. The branches of the mustelid and mephitid lineages were treated as belonging to the ancestors of the families and their hypothesized distributions are according to previous ancestral range estimations (Koepfli et al. 2008, Sato et al. 2009). Reconstruction of potential ancestral area combinations and dispersal scenarios took into account realistic dispersal routes (e.g., allowing Eurasia to connect only with North America) and the geological history of the region (e.g., formation of the Panama Isthmus during the late Miocene and Pliocene; Weyl 1980, Almendra and Rogers 2012).

### Bioclimatic range modeling

Vouchered localities of occurrence for *Bassaricyon* used in our analyses were extracted from museum specimen labels, often as clarified by associated field notes and journals, and from definitive published accounts. Gazetteers published by Paynter (1982, 1993, 1997), Stephens and Taylor (1983, 1985), Fairchild and Handley (1966), Handley (1976), Voss (1988), and Voss et al. (2002) were especially helpful in georeferencing Neotropical expedition and collecting localities represented in museum collections.

We used Maximum Entropy Modeling (Maxent) (Phillips et al. 2006) to predict the geographic range of the geographic range of the four *Bassaricyon* species at broad scales based on vouchered localities (Appendix 2) and 20 environmental variables representing potential vegetation and climate. For potential vegetation we used the 15 major habitat types classified as ecological biomes (Olson et al. 2001). For climate we used 19 BIOCLIM variables representing annual trends, seasonality, and extremes in temperature and precipitation across Central and South America (derived from Hijmans et al. [2005] as described at http://www.worldclim.org/bioclim.htm). We used all vouchered specimen localities to train the final model (excluding published records based only on visual observations). We also tested overall performance by running 10 model iterations while randomly withholding 20% of the points as test locations. To produce geographic ranges showing presence/absence of a species we used the average equal training sensitivity and specificity for the 10 test models as our probability cutoff value (Phillips et al. 2006).

## Results

### Phylogenetics

With the largest molecular sampling effort to date, we show that *Bassaricyon* is well resolved as a monophyletic genus (cf. Nyakatura and Bininda-Emonds 2012) within the family Procyonidae. All of our analyses resolve *Bassaricyon* as a clade with bootstrap and probability values of 100%. The sister genus to *Bassaricyon* is *Nasua*, a relationship consistently recovered in our analyses with 100% support. The divergence between *Bassaricyon* and *Nasua* was estimated at 10.2 million years old (mya) (95% Confidence Interval [CI] = 7.6 – 12.7 mya), consistent with previously published results (Koepfli et al. 2007, Eizirik et al. 2010).

**Figure 1.**
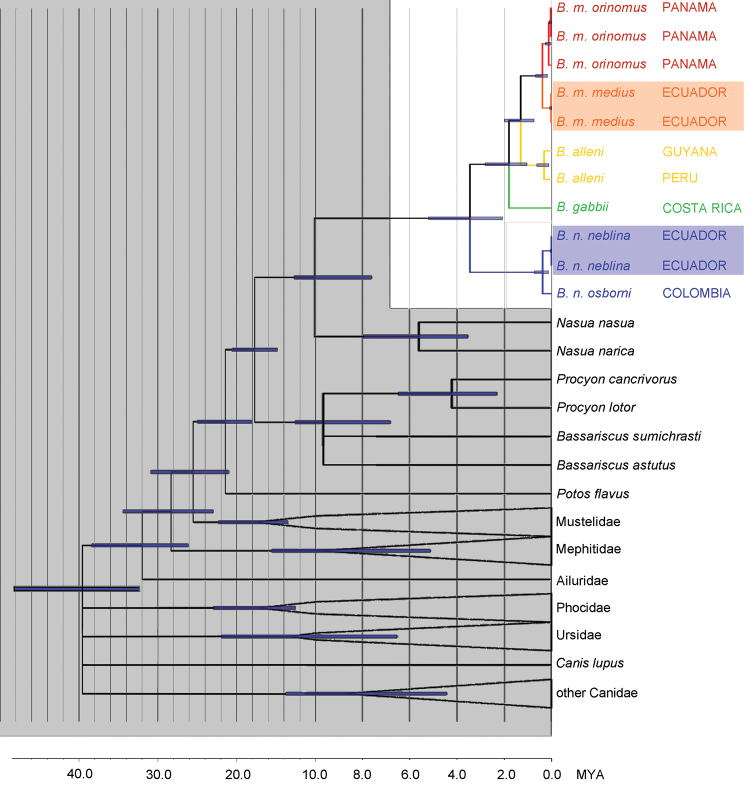
Phylogeny of the genus *Bassaricyon*. Phylogeny generated from the concatenated *CHRNA1* and cytochrome *b* sequences. All analyses consistently recovered the same relationships with high support. Divergence dating was generated in BEAST; bars show the 95% confidence interval at each node. Branches without support are collapsed and outgroup clades have been collapsed, leaving monophyletic groupings with 100% support. Data for *CHRNA1* are missing for *Bassaricyon gabbii*, for which DNA was extracted from a museum skull. All nodes in *Bassaricyon* have 1.00 Bayesian posterior probability, except the split between *Bassaricyon gabbii* and *Bassaricyon alleni*/*Bassaricyon medius* (0.97 Bayesian posterior probability). Non-focal and outgroup taxa are shaded in gray, *Bassaricyon* species and subspecies are color coded, samples of *Bassaricyon medius medius* and *Bassaricyon neblina neblina* that were collected within 5 km of each other in Ecuador are shaded.

The family Procyonidae is well resolved as monophyletic (100% bootstrap and probability values) with a divergence date of 21.4 mya (CI 18.1 – 25.0 mya), in agreement with the divergence estimate of 22.6 mya (CI 19.4 – 25.5 mya) by Eizirik et al. (2010). Eizirik et al. (2010) had a more constrained confidence interval on the age of this divergence, due to the incorporation of genes that are more informative at deeper nodes in the tree. We chose *CHRNA1* and cytochrome *b* with a focus toward resolving relationships within *Bassaricyon*; these markers are far more useful for determining relationships in recent radiations within Procyonidae than the deeper relationships within Carnivora. The only part of the Procyonidae where *CHRNA1* and cytochrome *b* did not provide sufficient resolution to re-construct recently published multi-gene topologies (Koepfli et al. 2007, Eizirik et al. 2010) was the divergence between the two species of *Bassariscus*, and *Procyon*. In our BEAST chronogram the divergence for *Bassariscus* is 7.6 mya (CI 4.8 – 10.6 mya) but the branch leading to their divergence has no support, and therefore is collapsed in the phylogeny (Figure 1; see also Koepfli et al. 2007, Eizirik et al. 2010). The other procyonid genera are well-supported monophyletic groups; according to our chronogram *Procyon lotor* and *Procyon cancrivorus* diverged 4.2 mya (CI 2.3 – 6.5 mya) and *Nasua narica* and *Nasua nasua* diverged 5.6 mya (CI 3.5 – 7.9 mya).

The concordance of our recovered topology and estimates of genetic divergence with previous phylogenetic studies of the Procyonidae suggests that data from cytochrome *b* and *CHRNA1* across sampled taxa have provided a well-supported framework in which the species relationships and divergence dates within *Bassaricyon* can be reliably assessed. Previous molecular phylogenetic studies have included either only one species (e.g., Ledje and Arnason 1996a, 1996b, and further studies using the same sequences, see below), identified as “*Bassaricyon gabbii*” (Genbank identifier X94931), but actually representing *Bassaricyon neblina* sp. n.; or, two species (Koepfli et al. 2007), identified as *Bassaricyon alleni* (correctly, sample from Amazonian Peru) and “*Bassaricyon gabbii*” (actually *Bassaricyon medius orinomus*,from Panama). Koepfli et al. (2007) gave the divergence estimate for these latter two taxa (i.e. *Bassaricyon alleni* and *Bassaricyon medius orinomus*) as 2.5–2.8 mya (CI 1.2–5.0 mya). Our results indicate that the earliest divergence within *Bassaricyon*, corresponding to the split between the ancestors of *Bassaricyon neblina* sp. n. and other *Bassaricyon*, occurred 3.5 mya (CI = 2.1 – 5.2 mya). Sequence divergence in cytochrome *b* between *Bassaricyon neblina* sp. n. and other *Bassaricyon* (including specimens of *Bassaricyon medius medius* collected in regional sympatry with *Bassaricyon neblina* sp. n. in the Western Andes of Ecuador) is 9.6-11.3% (Table 2). Cytochrome *b* sequence divergences between *Bassaricyon gabbii*, *Bassaricyon medius*, and *Bassaricyon alleni* are 6-7% (Table 2). Subspecific distances (see Systematics, below, for discussion of subspecies boundaries) are 1.6-2.0% within *Bassaricyon medius* (between *Bassaricyon medius medius* and *Bassaricyon medius orinomus*) and 1.6% within *Bassaricyon neblina* sp. n. (between *Bassaricyon neblina neblina* subsp. n. and *Bassaricyon neblina osborni* subsp. n., the two subspecies for which we have molecular data).

**Table 2.** Percentage sequence divergence in cytochrome *b* sequences (Kimura 2-Parameter) among specimens of *Bassaricyon* (numbers 1-11) and other Procyonidae (numbers 12-18) in our analyses (see Table 1, Figure 1). Numbers across the top row match numbered samples in the vertical column.

	1	2	3	4	5	6	7	8	9	10	11	12	13	14	15	16	17
1. *Bassaricyon medius orinomus* (Panama)																	
2. *Bassaricyon medius orinomus* (Panama)	0.2																
3. *Bassaricyon medius orinomus* (Panama)	0.3	0.4															
4. *Bassaricyon medius medius* (Ecuador)	1.9	1.9	1.6														
5. *Bassaricyon medius medius* (Ecuador)	1.9	2.0	1.6	0.1													
6. *Bassaricyon alleni* (Guyana)	6.9	7.0	6.6	7.2	7.4												
7. *Bassaricyon alleni* (Peru)	6.3	6.4	6.0	6.3	6.5	1.3											
8. *Bassaricyon gabbii* (Costa Rica)	7.3	7.1	7.0	6.9	6.7	6.3	6.6										
9. *Bassaricyon neblina neblina* (Ecuador)	10.1	10.1	9.8	10.4	10.6	11.3	11.0	9.9									
10. *Bassaricyon neblina neblina* (Ecuador)	10.1	10.1	9.8	10.5	10.6	11.3	11.0	9.9	0.0								
11. *Bassaricyon neblina osborni* (Colombia)	10.0	9.9	9.6	10.3	10.4	11.2	10.6	10.4	1.6	1.6							
12. *Potos flavus*	28.7	28.9	28.7	29.5	29.5	29.8	29.0	28.1	29.8	29.9	28.9						
13. *Procyon lotor*	34.8	34.3	34.3	35.2	34.9	35.6	34.9	33.0	33.8	33.7	32.7	27.3					
14. *Procyon cancrivorus*	31.9	31.2	31.2	32.2	32.0	32.1	29.9	31.9	32.0	31.8	30.4	29.4	13.1				
15. *Bassariscus astutus*	30.7	30.5	30.0	29.8	30.0	30.8	30.0	29.4	29.3	29.1	29.5	29.6	20.7	17.8			
16. *Bassariscus sumichrasti*	28.1	27.4	27.7	27.7	27.9	27.7	25.7	28.3	26.2	26.1	25.6	26.8	17.1	18.3	15.8		
17. *Nasua nasua*	26.8	26.7	26.7	28.1	28.4	25.4	24.1	25.7	25.0	24.8	24.1	35.6	35.8	30.3	30.5	29.1	
18. *Nasua narica*	30.3	29.7	30.0	30.2	30.0	29.0	29.2	28.8	25.1	25.1	24.2	31.3	29.7	26.4	27.3	26.3	20.4

We obtained the highest bootstrap and posterior probability support values (100% and 1.0 respectively) for relationships within *Bassaricyon* with every method of phylogenetic inference that was used in this study. The single exception was that the topology that recovered the node uniting *Bassaricyon alleni* and *Bassaricyon medius* to the exclusion of *Bassaricyon gabbii* was assigned a slightly lower Bayesian posterior probability value of 0.97, but all other methods lent full support to this topology (*Bassaricyon gabbii*,(*Bassaricyon medius*, *Bassaricyon alleni*)). These results were also well-supported by our comparisons of morphological characters and together lend strong support for this scenario as being an accurate representation of the evolutionary history of diversification within *Bassaricyon*.

### Biogeography

**Figure 2.**
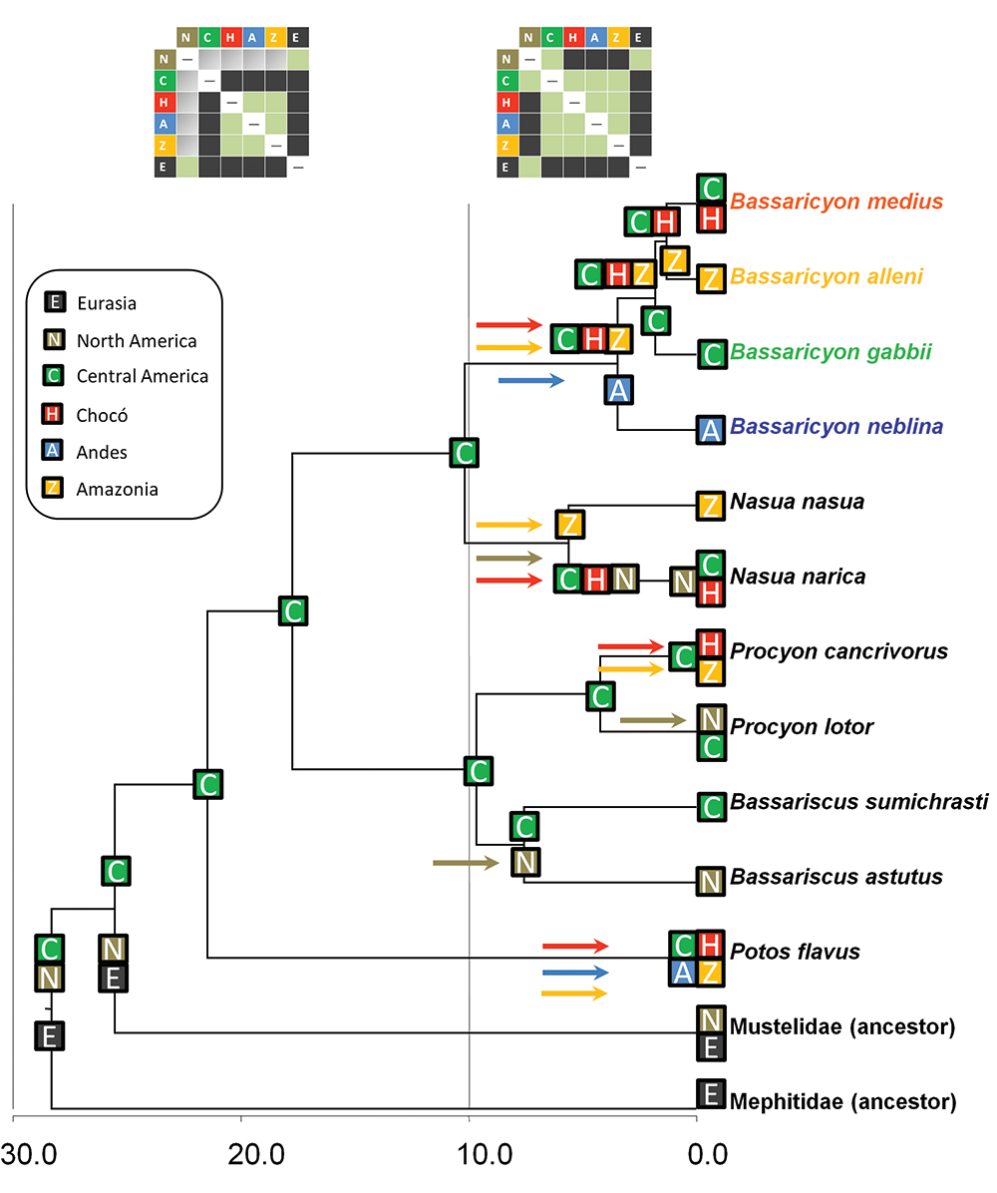
Historical biogeography of procyonids. Reconstructed under the DEC model implemented in Lagrange. See legend for geographical areas used in the analysis. Colored squares at the tip of the branches reflect the distribution of taxa, and previously inferred distributions of the ancestors of mustelids and mephitids. Colored squares at the nodes represent the geographic ranges with the highest probabilites in the DEC model inherited by each descendant branch. Colored arrows reflect dispersal events between ancestral and derived areas, with colors matching with recipient areas. Upper boxes: different dispersal constraints at time intervals 0–10 mya and 10–30 mya, the former to simulate the effect of the land bridge formation between Central and South America, the latter restricted dispersal due to the absence of the land bridge; the cells in green indicate no restriction to dispersal, cells in gray indicate a reduction by half in dispersal capability, and cells in black do not allow dispersal. Timescale in millions of years before present (mya).

The historical biogeographic reconstruction for the Procyonidae using the DEC model sets Central America as the likely center of origin of crown-group procyonids (Figure 2) (though we note that the family has many extinct, Eocene to Miocene representatives in North America and Europe). Major splits within the family appear to have occurred in Central America previous to the formation of the Panamanian Isthmus, and all the dispersal events resulting in the extant species have occurred within the last 10 million years. All those dispersal events involving southward movements seem to have occurred up to *circa* 6 mya, coinciding with the initial uplift of the Panamanian Isthmus, and, presumably once it was consolidated, with the Great American Biotic Interchange (GABI) (Figures 1–2). The clade containing all olingo species is likely to have originated directly as a result of the formation of the Panamanian Isthmus, and provides evidence of a complex pattern of dispersal events out of Central America (Figure 2).

### Morphology and morphometrics

**Figure 3.**
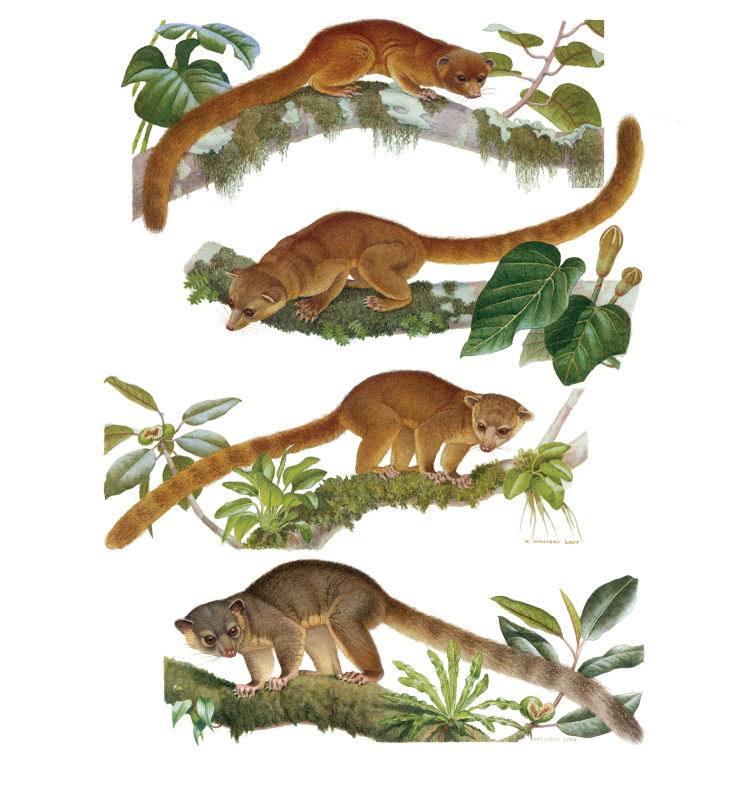
Illustrations of the species of *Bassaricyon*. From top to bottom, *Bassaricyon neblina* sp. n. (*Bassaricyon neblina ruber* subsp. n. of the western slopes of the Western Andes of Colombia), *Bassaricyon medius* (*Bassaricyon medius orinomus* of eastern Panama), *Bassaricyon alleni* (Peru), and *Bassaricyon gabbii* (Costa Rica, showing relative tail length longer than average). Artwork by Nancy Halliday.

**Figure 4.**
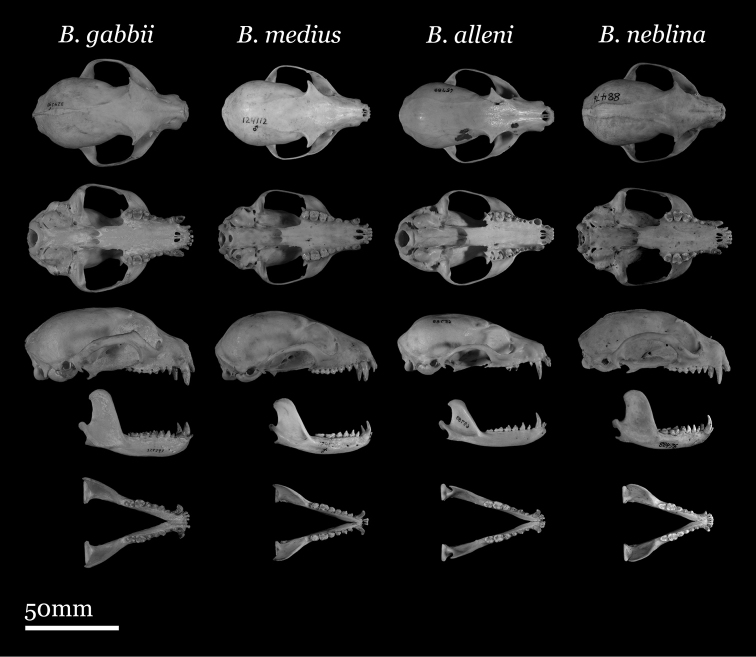
Skulls of adult male *Bassaricyon*. From left to right: *Bassaricyon gabbii* (USNM 324293, Cerro Punta, 1700 m, Chiriqui Mountains, Panama); *Bassaricyon medius medius* (MVZ 124112, Dagua, 1800 m, Colombia); *Bassaricyon alleni* (FMNH 65789, Chanchamayo, 1200 m, Junin, Peru); *Bassaricyon neblina osborni* (FMNH 88476, Munchique, 2000 m, Cauca, Colombia). Scale bar = 50 mm.

**Figure 5.**
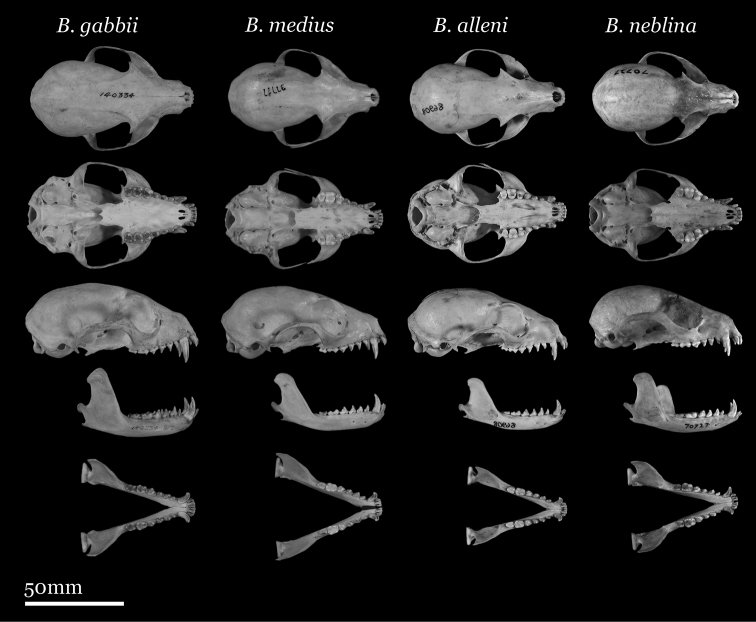
Skulls of adult female *Bassaricyon*. From left to right: *Bassaricyon gabbii* (AMNH 140334, Lajas Villa, Costa Rica); *Bassaricyon medius orinomus* (AMNH 37797, Puerta Valdivia, Antioquia District, Colombia); *Bassaricyon alleni* (FMNH 86908, Santa Rita, Rio Nanay, Maynas, Loreto Region, Peru); *Bassaricyon neblina hershkovitzi* (FMNH 70727, San Antonio, Agustin, Huila District, Colombia). Scale bar = 50 mm.

Our study of *Bassaricyon* taxonomy originally began with close examination of craniodental traits of museum specimens, which quickly revealed to the first author the existence of *Bassaricyon neblina* sp. n., which is highly distinctive morphologically. Close examinations of skins and skulls revealed clear differences in qualitative traits, and in external and craniodental measurements and proportions, between the four principal *Bassaricyon* lineages identified in this paper (which we recognize taxonomically as *Bassaricyon neblina* sp. n., *Bassaricyon gabbii*, *Bassaricyon alleni*, and *Bassaricyon medius*; Figures 3–5). Externally, these especially include differences in body size, pelage coloration, pelage length, relative length of the tail, and relative size of the ears (Figure 3, Table 5). Craniodentally, these especially include differences in skull size, relative size of the premolars and molars, configuration of molar cusps, relative size of the auditory bullae and external auditory meati, and the shape of the postdental palatal shelf (Figures 4–5, Tables 3–4). These and other differences are discussed in greater detail in the species accounts provided later in the paper.

**Table 3.** Cranial measurements for olingo species (compiled separately by sex). For each measurement, means are provided, ± standard deviation, with ranges in parentheses.

		*Bassaricyon gabbii* *n*= 11 ♂♂, 11 ♀♀	*Bassaricyon medius* *n*= 18 ♂♂, 27 ♀♀	*Bassaricyon alleni* *n*= 12 ♂♂, 17 ♀♀	*Bassaricyon neblina* *n*= 10 ♂♂, 9 ♀♀
CBL	**♂♂**	80.8 ± 1.50 (78.1 - 83.0)	79.4 ± 2.67 (74.5 - 85.1)	79.4 ± 1.81 (76.5 - 82.8)	74.5 ± 3.26 (70.1 - 79.5)
	**♀♀**	78.2 ± 1.75 (75.0 - 80.2)	77.3 ± 2.70 (70.8 - 82.3)	77.0 ± 2.24 (73.1 - 80.5)	75.1 ± 1.49 (72.9 - 77.9)
ZYG	**♂♂**	55.2 ± 2.76 (49.5 - 58.7)	52.0 ± 2.66 (48.3 - 56.7)	51.6 ± 1.02 (49.0 - 52.8)	50.1 ± 3.02 (46.2 - 54.4)
	**♀♀**	51.3 ± 1.90 (48.1 - 54.4)	50.0 ± 2.50 (44.4 - 54.0)	50.2 ± 0.99 (48.6 - 52.2)	49.0 ± 2.69 (44.6 - 53.0)
BBC	**♂♂**	36.1 ± 0.86 (34.3 - 37.6)	35.1 ± 1.16 (32.9 - 37.5)	35.4 ± 0.80 (34.2 - 36.8)	34.6 ± 1.62 (32.4 - 37.5)
	**♀♀**	35.7 ± 1.34 (33.1 - 37.5)	34.6 ± 1.20 (32.0 - 37.2)	34.9 ± 0.91 (33.3 - 36.8)	34.2 ± 1.62 (31.0 - 36.6)
HBC	**♂♂**	28.7 ± 0.88 (26.4 - 29.7)	27.6 ± 0.84 (26.5 - 29.3)	27.4 ± 0.73 (26.2 - 28.5)	27.4 ± 0.61 (26.5 - 28.3)
	**♀♀**	27.9 ± 0.74 (26.9 - 28.8)	26.9 ± 0.90 (25.4 - 28.5)	26.9 ± 0.63 (26.0 - 28.1)	26.5 ± 0.93 (24.9 - 27.8)
MTR	**♂♂**	28.5 ± 0.50 (27.8 - 29.3)	28.6 ± 0.87 (27.0 - 30.4)	28.4 ± 0.83 (26.5 - 29.5)	26.5 ± 1.38 (24.5 - 28.7)
	**♀♀**	27.3 ± 1.02 (26.0 - 29.0)	27.7 ± 0.90 (25.6 - 29.1)	27.3 ± 0.69 (26.1 - 28.5)	26.9 ± 0.88 (25.8 - 28.3)
CC	**♂♂**	18.7 ± 1.12 (17.2 - 20.4)	16.4 ± 0.92 (15.0 - 17.9)	16.8 ± 0.51 (15.8 - 17.6)	15.9 ± 0.94 (14.7 - 17.1)
	**♀♀**	16.9 ± 0.76 (15.6 - 17.9)	15.7 ± 0.80 (14.5 - 17.2)	15.9 ± 0.55 (14.8 - 16.8)	15.7 ± 0.47 (14.9 - 16.4)
WPP	**♂♂**	11.3 ± 1.27 (9.0 - 12.9)	10.3 ± 0.95 (8.4 - 12.1)	10.4 ± 0.82 (8.7 - 11.8)	11.7 ± 1.05 (10.6 - 14.0)
	**♀♀**	10.7 ± 0.99 (9.3 - 12.7)	10.3 ± 0.90 (9.0 - 13.0)	9.9 ± 0.89 (8.2 - 11.7)	11.6 ± 0.87 (10.5 - 12.7)
LPP	**♂♂**	12.3 ± 0.99 (10.7 - 14.0)	10.2 ± 0.88 (7.9 - 11.7)	10.8 ± 1.21 (9.3 - 12.9)	11.2 ± 1.24 (9.2 - 12.7)
	**♀♀**	10.8 ± 0.77 (9.7 - 12.0)	10.1 ± 0.90 (8.1 - 11.8)	10.4 ± 0.67 (8.7 - 11.6)	11.1 ± 0.82 (9.7 - 12.3)
LAB	**♂♂**	13.8 ± 0.63 (12.9 - 14.7)	14.0 ± 0.81 (12.8 - 15.6)	15.1 ± 0.76 (14.1 - 16.8)	11.8 ± 0.76 (10.9 - 13.3)
	**♀♀**	13.8 ± 0.67 (12.9 - 14.8)	14.0 ± 0.80 (12.6 - 15.2)	14.4 ± 0.81 (13.0 - 15.6)	12.2 ± 0.51 (11.0 - 12.7)
EAM	**♂♂**	3.6 ± 0.47 (2.6 - 4.2)	3.9 ± 0.33 (3.4 - 4.5)	3.8 ± 0.40 (3.2 - 4.5)	2.9 ± 0.22 (2.5 - 3.1)
	**♀♀**	3.6 ± 0.39 (3.0 - 4.2)	3.9 ± 0.30 (3.5 - 4.7)	3.8 ± 0.36 (3.2 - 4.4)	3.2 ± 0.33 (2.6 - 3.5)

**Table 4.** Selected dental measurements of olingo species. For each measurement, means are provided, ± standard deviation, with ranges in parentheses.

	*Bassaricyon gabbii* *n*= 22	*Bassaricyon medius* *n*= 45	*Bassaricyon alleni* *n*= 34	*Bassaricyon neblina* *n*= 19
p1 width	1.7 ± 0.17 (1.4 - 2.1)	1.7 ± 0.13 (1.4 - 2.0)	1.7 ± 0.12 (1.5 - 1.9)	1.6 ± 0.13 (1.4 - 1.8)
p2 width	2.4 ± 0.24 (2.0 - 2.8)	2.2 ± 0.18 (1.8 - 2.6)	2.2 ± 0.15 (1.9 - 2.5)	2.1 ± 0.17 (1.9 - 2.5)
p3 width	2.7 ± 0.21 (2.3 - 3.0)	2.5 ± 0.18 (2.2 - 2.9)	2.6 ± 0.16 (2.2 - 2.9)	2.4 ± 0.22 (2.1 - 2.9)
p4 width	3.4 ± 0.27 (3.0 - 3.9)	3.2 ± 0.18 (2.8 - 3.6)	3.4 ± 0.21 (2.8 - 3.7)	3.3 ± 0.15 (3.0 - 3.7)
P2 width	2.4 ± 0.24 (2.1 - 2.9)	2.3 ± 0.19 (1.9 - 2.8)	2.2 ± 0.17 (1.9 - 2.7)	2.1 ± 0.19 (1.8 - 2.5)
P3 width	2.9 ± 0.22 (2.5 - 3.3)	3.0 ± 0.29 (2.5 - 3.6)	3.0 ± 0.22 (2.6 - 3.5)	2.9 ± 0.21 (2.6 - 3.4)
P4 length	4.4 ± 0.24 (3.9 - 4.8)	4.2 ± 0.27 (3.6 - 4.9)	4.2 ± 0.20 (3.8 - 4.6)	4.5 ± 0.24 (4.1 - 4.9)
P4 width	5.1 ± 0.35 (4.5 - 5.6)	4.7 ± 0.26 (4.2 - 5.4)	4.8 ± 0.23 (4.4 - 5.6)	5.0 ± 0.40 (4.5 - 5.9)
M1 length	5. 0 ± 0.27 (4.4 - 5.4)	5.0 ± 0.29 (4.3 - 5.6)	5.1 ± 0.21 (4.6 - 5.5)	5.3 ± 0.35 (4.8 - 6.1)
M1 width	5.5 ± 0.30 (4.7 - 5.9)	5.3 ± 0.32 (4.7 - 5.9)	5.5 ± 0.28 (4.9 - 6.0)	5.8 ± 0.31 (5.4 - 6.4)
M2 length	3.7 ± 0.32 (2.8 - 4.1)	4.0 ± 0.25 (3.2 - 4.4)	3.8 ± 0.27 (3.3 - 4.4)	3.8 ± 0.35 (3.3 - 4.4)
M2 width	4.6 ± 0.38 (4.0 - 5.3)	4.7 ± 0.27 (4.1 - 5.2)	4.7 ± 0.28 (4.0 - 5.2)	4.8 ± 0.24 (4.4 - 5.4)
m1 length	5.6 ± 0.31 (5.0 - 6.3)	5.7 ± 0.26 (4.9 - 6.2)	5.6 ± 0.22 (5.2 - 6.0)	5.8 ± 0.29 (5.4 - 6.3)
m1 width	4.3 ± 0.29 (3.8 - 4.9)	4.3 ± 0.21 (3.9 - 4.7)	4.3 ± 0.23 (3.7 - 4.8)	4.8 ± 0.22 (4.5 - 5.3)
m2 length	4.8 ± 0.25 (4.4 - 5.3)	5.1 ± 0.36 (4.2 - 5.7)	4.8 ± 0.25 (4.4 - 5.4)	5.0 ± 0.35 (4.4 - 5.6)
m2 width	3.8 ± 0.24 (3.3 - 4.2)	3.7 ± 0.24 (3.2 - 4.2)	3.7 ± 0.19 (3.3 - 4.0)	3.8 ± 0.17 (3.5 - 4.1)

**Table 5.** External measurements of olingo species. For each measurement, means are provided, ± standard deviation, with ranges in parentheses.

	*Bassaricyon gabbii* *n*= 13	*Bassaricyon medius* *n*= 36	*Bassaricyon alleni* *n*= 27	*Bassaricyon neblina* *n*= 19
TL	873 ± 54.8 (785 - 970)	819 ± 60.5 (680 - 905)	842 ± 50.6 (705 - 985)	745 ± 33.7 (660 - 820)
Tail	445 ± 40.3 (400 - 521)	441 ± 44.6 (350 - 520)	450 ± 28.8 (401 - 530)	390 ± 21 (335 - 424)
HF	84 ± 8.7 (65 - 100)	81 ± 7.3 (58 - 92)	81 ± 5.8 (70 - 92)	76 ± 6.9 (60 - 86)
Ear	36 ± 4.7 (25 - 44)	37 ± 5.4 (25 - 44)	37 ± 3.4 (30 - 43)	34 ± 4.3 (25 - 39)
Mass (g)	1382 ± 165 (1136 - 1580)	1076 ± 71.6 (915 - 1200)	1336 ± 152 (1100 - 1500)	872 ± 169 (750 - 1065)
HB	428 ± 27.9 (373 - 470)	379 ± 23.2 (310 - 415)	391 ± 29.3 (304 - 455)	355 ± 21.1 (325 - 400)
Tail/HB	1.04 ± 0.1 (0.9 - 1.2)	1.16 ± 0.1 (1.0 - 1.4)	1.15 ± 0.08 (1.0 - 1.3)	1.10 ± 0.08 (1.0 - 1.2)

**Figure 6.**
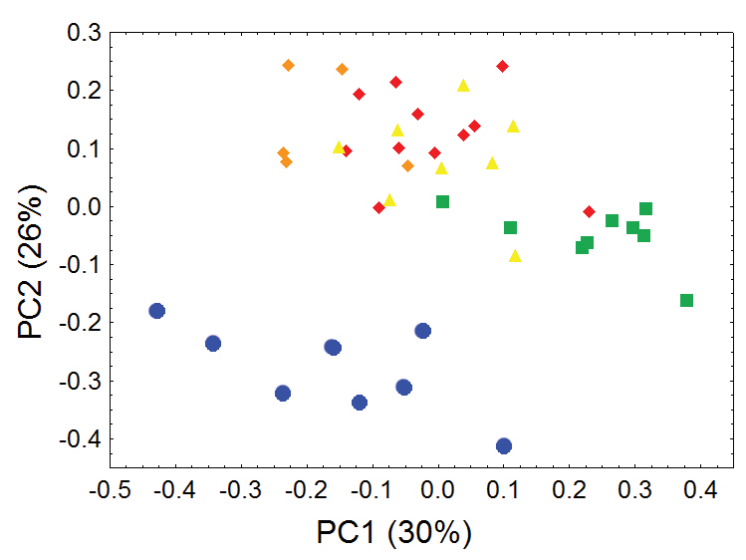
Morphometric distinction between Olinguitos and other *Bassaricyon*, males. Morphometric dispersion (first two components of a principal component analysis) of 41 adult male *Bassaricyon* skulls based on 21 craniodental measurements (see Appendix 1, Table A1). The most notable morphometric distinction is between the Olinguito (blue circles) and all other *Bassaricyon* taxa. The plot also demonstrates substantial morphometric variability across geographic populations of the Olinguito, which we characterize with the description of four subspecies across different Andean regions. Symbols: blue circles (*Bassaricyon neblina*), green squares (*Bassaricyon gabbii*), yellow triangles (*Bassaricyon alleni*), orange diamonds (*Bassaricyon medius medius*), red diamonds (*Bassaricyon medius orinomus*).

**Figure 7.**
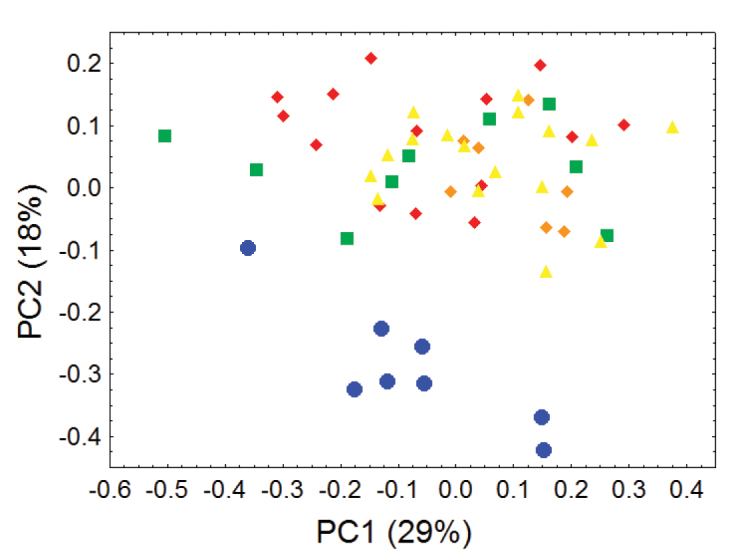
Morphometric distinction between Olinguitos and other *Bassaricyon*, females. Morphometric dispersion (first two components of a principal component analysis) of 55 adult female *Bassaricyon* skulls based on 24 craniodental measurements (see Appendix 1, Table A2). The most notable morphometric distinction is between the Olinguito (blue circles) and all other *Bassaricyon* taxa. The plot also demonstrates substantial morphometric variability across geographic populations of the Olinguito, which we characterize with the description of four subspecies across different Andean regions. Symbols: blue circles (*Bassaricyon neblina*), green squares (*Bassaricyon gabbii*), yellow triangles (*Bassaricyon alleni*), orange diamonds (*Bassaricyon medius medius*), red diamonds (*Bassaricyon medius orinomus*).

Principal component analyses of cranial and dental measurements support our molecular results in clearly identifying a fundamental morphometric separation between the Olinguito (*Bassaricyon neblina* sp. n.) and all other *Bassaricyon* taxa, in separate comparisons involving both males and females (Figures 6–7, Appendix 1). When first and second principal components are juxtaposed in a bivariate plot, Olinguito specimens demonstrate clear morphometric separation from all other *Bassaricyon*, despite overlap between these clusters in body size (as indicated by overlap in the first principal component, on which all or most variables in the analysis show positive [males] or negative [females] loadings). Despite smaller average body size compared to other *Bassaricyon*, the morphometric distinctness of Olinguito specimens is reflected not especially in small size but rather primarily by separation along the second principal component, indicating trenchant differences in overall shape and proportion, especially reflecting consistent differences in the molars, auditory bullae, external auditory meati, and palate, in which the Olinguito differs strongly and consistently from other *Bassaricyon* (Figures 6–7, Tables 3–4, Appendix 1).

**Figure 8.**
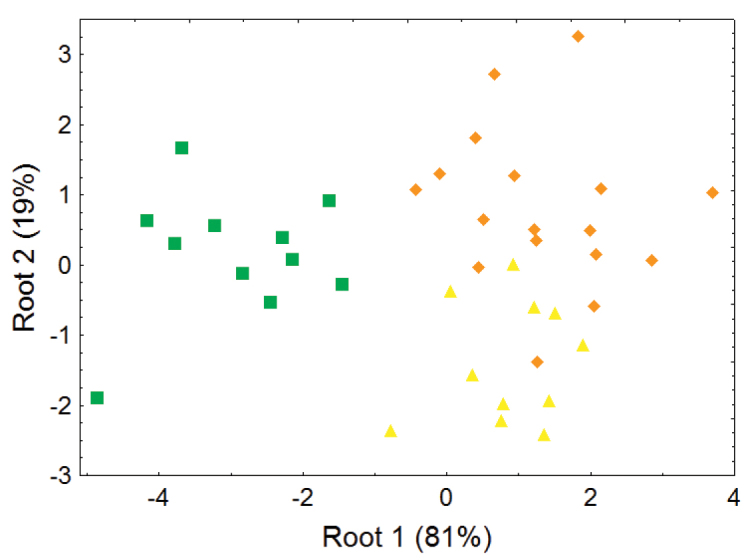
Morphometric distinction between species of *Bassaricyon*, excluding the Olinguito, adult males. Morphometric dispersion (first two variates of a discriminant function analysis) of 39 adult male *Bassaricyon* skulls based on 8 craniodental measurements (see Appendix 1, Table A3). Symbols: green squares (*Bassaricyon gabbii*), yellow triangles (*Bassaricyon alleni*), orange diamonds (*Bassaricyon medius*).

The lower elevation olingo taxa *Bassaricyon gabbii*, *Bassaricyon medius*, and *Bassaricyon alleni* are not separable in most principal component analyses of craniodental measurements (e.g., Figures 6–7), but discriminant function analyses of craniodental measurements (e.g., Figure 8, showing separation of male skulls) separates them into discrete clusterings with few misclassifications, and identifies some of the more important craniodental traits that help to distinguish between them (Appendix 1). These (and other, qualitative) craniodental distinctions are complemented by differences in pelage features and genetic divergences that we discuss below.

**Figure 9.**
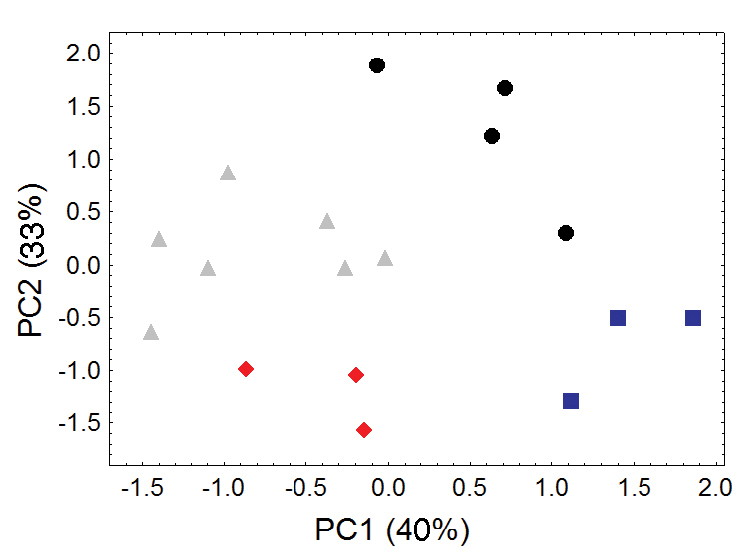
Morphometric distinction between Olinguito subspecies. Both sexes combined. Morphometric dispersion (first two components of a principal component analysis) of 17 adultskulls based on 13 cranial measurements (see Appendix 1, Table A4). (Dental measurements also discretely partition these subspecies in a separate principal component analysis, not shown.) Black dots = *Bassaricyon neblina neblina*; gray triangles = *Bassaricyon neblina osborni*; red diamonds = *Bassaricyon neblina ruber*; blue squares = *Bassaricyon neblina hershkovitzi*.

**Figure 10.**
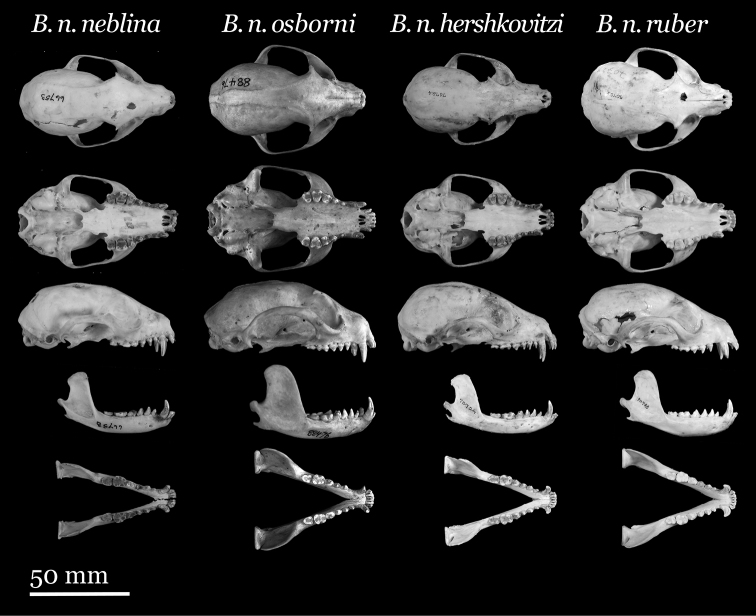
Skulls of Olinguito subspecies. From left to right: *Bassaricyon neblina neblina* (AMNH 66753, holotype, old adult female, Las Maquinas, Ecuador); *Bassaricyon neblina osborni* (FMNH 88476, holotype, adult male, Munchique, 2000 m, Cauca Department, Colombia); *Bassaricyon neblina hershkovitzi* (FMNH 70724, paratype, adult male, San Antonio, Agustin, Huila District, Colombia); *Bassaricyon neblina ruber* (FMNH 70723, paratype, adult male, Guapantal, 2200 m, Urrao, Antioquia Department, Colombia). Scale bar = 50 mm.

Because of marked and consistent differences in body size between the two regional populations of *Bassaricyon medius* (one distributed in western South America, the other primarily distributed in Panama), we choose to recognize these two as separate subspecies (*Bassaricyon medius medius* and *Bassaricyon medius orinomus*, respectively, Tables 6–7). The Olinguito likewise shows a clear pattern of geographic variation, with different regional populations in the Northern Andes showing consistent differences in craniodental size and morphology (Figures 9–10, Table 8, Appendix 1), as well as pelage coloration and length. We recognize four distinctive subspecies of the Olinguito throughout its recorded distribution, as discussed in the description of *Bassaricyon neblina* sp. n., below. Two of these subspecies are included in our genetic comparisons; genetic comparisons involving the remaining two subspecies remain a goal for the future.

**Table 6.** Cranial measurements for the two subspecies of *Bassaricyon medius*. For each measurement, means are provided, ± standard deviation, with ranges in parentheses.

		*Bassaricyon medius medius*	*Bassaricyon medius orinomus*
		W Colombia, W Ecuador	C Panama to N Colombia
		n = 5 ♂♂, 7 ♀♀	n = 12 ♂♂, 17 ♀♀
CBL	**♂♂**	77.2 ± 1.81 (74.5 - 78.8)	80.3 ± 2.50 (76.2 - 85.1)
	**♀♀**	75.4 ± 1.65 (72.4 - 76.7)	78.8 ± 1.72 (75.5 - 82.3)
ZYG	**♂♂**	50.2 ± 1.14 (48.9 - 51.2)	53.0 ± 2.57 (48.9 - 56.7)
	**♀♀**	48.5 ± 1.69 (46.5 – 51.0)	51.2 ± 1.98 (47.4 – 54.0)
BBC	**♂♂**	34.0 ± 0.80 (32.9 - 34.8)	35.6 ± 0.98 (34.0 - 37.5)
	**♀♀**	34.4 ± 0.41 (33.7 – 35.0)	35.0 ± 1.15 (32.8 - 37.2)
HBC	**♂♂**	28.2 ± 1.06 (27.1 - 29.3)	27.4 ± 0.62 (26.6 - 28.3)
	**♀♀**	26.8 ± 0.89 (26.1 - 28.5)	27.0 ± 0.89 (25.4 - 28.5)
MTR	**♂♂**	28.5 ± 0.97 (27.3 - 29.8)	28.7 ± 0.90 (27.0 - 30.4)
	**♀♀**	27.1 ± 0.78 (25.6 - 27.9)	28.0 ± 0.77 (26.4 - 29.1)
CC	**♂♂**	15.9 ± 0.69 (15.1 - 17.0)	16.7 ± 0.94 (15.0 - 17.9)
	**♀♀**	15.0 ± 0.46 (14.5 - 15.8)	16.1 ± 0.71 (14.6 - 17.2)
WPP	**♂♂**	9.7 ± 0.95 (8.4 - 10.8)	10.6 ± 0.91 (8.6 - 12.1)
	**♀♀**	10.0 ± 0.57 (9.1 - 10.6)	10.3 ± 1.04 (9.0 - 13.0)
LPP	**♂♂**	9.4 ± 1.03 (7.9 - 10.6)	10.5 ± 0.64 (9.8 - 11.7)
	**♀♀**	9.8 ± 0.84 (8.9 - 11.3)	10.2 ± 1.01 (8.1 - 11.8)
LAB	**♂♂**	13.6 ± 0.72 (12.8 - 14.6)	14.2 ± 0.84 (13.1 - 15.6)
	**♀♀**	13.4 ± 0.45 (12.6 - 13.9)	14.3 ± 0.73 (12.8 - 15.2)
EAM	**♂♂**	3.9 ± 0.47 (3.4 - 4.5)	3.9 ± 0.27 (3.5 - 4.4)
	**♀♀**	3.9 ± 0.34 (3.5 - 4.4)	3.9 ± 0.28 (3.6 - 4.7)

**Table 7.** External measurements for the two subspecies of *Bassaricyon medius*. For each measurement, means are provided, ± standard deviation, with ranges in parentheses.

	*Bassaricyon medius medius* W Colombia, W Ecuador *n*= 12	*Bassaricyon medius orinomus* C Panama to N Colombia *n*= 24
**TL**	754 ± 49.7 (680 - 819)	844 ± 42.9 (770 - 905)
**Tail**	392 ± 29.1 (350 - 435)	460 ± 33.6 (400 - 520)
**HF**	73 ± 5.4 (58 - 79)	85 ± 3.5 (77 - 92)
**Ear**	32 ± 4.8 (25 - 40)	39 ± 4 (30 - 44)
**Mass (g)**	1058 ± 146 (915 - 1200)	1090 ± 19.2 (1050 - 1100)
**HB**	362 ± 29.5 (310 - 415)	385 ± 17.2 (355 - 410)
**Tail/HB**	1.1 ± 0.09 (0.97 - 1.24)	1.2 ± 0.08 (1.04 - 1.35)

**Table 8.** Dental and cranial measurements of Olinguito (*Bassaricyon neblina*) subspecies. For each measurement, means are provided, ± standard deviation, with ranges in parentheses.

	*Bassaricyon neblina ruber* *n*= 3	*Bassaricyon neblina hershkovitzi* *n*= 4	*Bassaricyon neblina osborni* *n*= 8	*Bassaricyon neblina neblina* *n*= 4
p1 width	1.4 ± 0.06 (1.4 - 1.5)	1.5 ± 0.12 (1.4 - 1.6)	1.6 ± 0.09 (1.6 - 1.8)	1.7 ± 0.11 (1.5 - 1.8)
p2 width	2.1 ± 0.14 (1.9 - 2.2)	1.9 ± 0.06 (1.9 – 2.0)	2.2 ± 0.15 (2.0 - 2.5)	2.2 ± 0.17 (2.1 - 2.4)
p3 width	2.4 ± 0.08 (2.3 - 2.5)	2.2 ± 0.06 (2.1 - 2.2)	2.5 ± 0.16 (2.4 - 2.8)	2.4 ± 0.32 (2.2 - 2.9)
p4 width	3.3 ± 0.11 (3.2 - 3.4)	3.1 ± 0.12 (3.0 - 3.3)	3.4 ± 0.13 (3.2 - 3.7)	3.4 ± 0.09 (3.3 - 3.5)
P2 width	2.0 (2.0 – 2.0)	1.9 ± 0.05 (1.8 – 2.0)	2.2 ± 0.17 (2.1 - 2.5)	2.3 ± 0.15 (2.2 - 2.5)
P3 width	2.9 ± 0.17 (2.7 - 3.1)	2.7 ± 0.10 (2.6 - 2.8)	3.0 ± 0.19 (2.8 - 3.4)	3.1 ± 0.15 (2.9 - 3.3)
P4 length	4.3 ± 0.21 (4.1 - 4.5)	4.2 ± 0.13 (4.1 - 4.3)	4.5 ± 0.17 (4.3 - 4.8)	4.7 ± 0.17 (4.5 - 4.9)
P4 width	4.6 ± 0.14 (4.5 - 4.8)	5.0 ± 0.23 (4.8 - 5.3)	4.9 ± 0.20 (4.6 - 5.1)	5.7 ± 0.13 (5.6 - 5.9)
M1 length	5.0 ± 0.12 (5.0 - 5.2)	5.0 ± 0.25 (4.8 - 5.4)	5.3 ± 0.23 (5.0 - 5.6)	5.7 ± 0.4 (5.2 - 6.1)
M1 width	5.5 ± 0.14 (5.4 - 5.6)	5.5 ± 0.10 (5.4 - 5.6)	5.8 ± 0.20 (5.5 - 6.1)	6.2 ± 0.13 (6.1 - 6.4)
M2 length	3.6 ± 0.22 (3.5 - 3.9)	3.5 ± 0.16 (3.3 - 3.7)	4.1 ± 0.29 (3.6 - 4.4)	3.9 ± 0.4 (3.3 - 4.2)
M2 width	4.5 ± 0.13 (4.4 - 4.6)	4.7 ± 0.03 (4.7 - 4.8)	4.8 ± 0.20 (4.6 - 5.2)	4.9 ± 0.3 (4.7 - 5.4)
m1 length	5.5 ± 0.05 (5.4 - 5.5)	5.8 ± 0.21 (5.6 – 6.0)	5.8 ± 0.18 (5.6 – 6.0)	6.2 ± 0.03 (6.2 - 6.3)
m1 width	4.7 ± 0.12 (4.6 - 4.8)	4.8 ± 0.17 (4.7 – 5.0)	4.8 ± 0.26 (4.5 - 5.3)	5.0 ± 0.22 (4.7 - 5.2)
m2 length	4.7 ± 0.39 (4.4 - 5.1)	5.0 ± 0.37 (4.5 - 5.2)	5.2 ± 0.26 (4.9 - 5.6)	4.8 ± 0.22 (4.5 - 5.1)
m2 width	3.7 ± 0.09 (3.6 - 3.8)	3.7 ± 0.19 (3.5 - 3.9)	3.9 ± 0.10 (3.7 - 4.0)	3.9 ± 0.16 (3.7 - 4.1)
CBL	73.0 ± 0.58 (72.4 - 73.5)	71.4 ± 1.13 (70.1 - 72.9)	76.6 ± 1.64 (75.1 - 79.5)	75.9 ± 1.4 (74.6 - 77.9)
ZYG	51.1 ± 2.28 (48.9 - 53.4)	46.7 ± 0.60 (46.2 - 47.5)	51.7 ± 1.73 (49.1 - 54.4)	46.9 ± 1.59 (44.6 - 48)
BBC	36.0 ± 1.44 (34.7 - 37.5)	32.9 ± 0.54 (32.4 - 33.6)	35.1 ± 0.90 (33.9 - 36.6)	33.2 ± 1.62 (31.0 - 34.9)
HBC	27.7 ± 0.55 (27.2 - 28.3)	27.6 ± 0.38 (27.1 - 27.9)	27.2 ± 0.58 (26.5 - 28.2)	25.8 ± 0.63 (24.9 - 26.2)
MTR	25.9 ± 0.22 (25.7 - 26.1)	25.1 ± 0.56 (24.5 - 25.8)	27.4 ± 0.78 (26.0 - 28.7)	27.5 ± 0.56 (27 - 28.3)
CC	15.7 ± 0.52 (15.4 - 16.3)	14.9 ± 0.15 (14.7 – 15.0)	16.4 ± 0.54 (15.5 - 17.1)	15.6 ± 0.25 (15.4 - 15.9)
WPP	12.1 ± 0.25 (11.8 - 12.3)	11.8 ± 1.54 (10.6 – 14.0)	11.8 ± 0.74 (10.8 - 12.8)	10.9 ± 0.8 (10.5 - 12.1)
LPP	10.9 ± 0.54 (10.3 - 11.4)	9.7 ± 0.34 (9.2 - 9.9)	11.9 ± 0.56 (11.0 - 12.7)	11.2 ± 1.05 (9.7 - 12.3)
LAB	11.7 ± 0.38 (11.4 - 12.1)	11.2 ± 0.40 (10.9 - 11.8)	12.3 ± 0.60 (11.2 - 13.3)	12.5 ± 0.18 (12.3 - 12.7)
EAM	2.7 (2.7 - 2.7)	3.2 ± 0.16 (3.1 - 3.4)	2.9 ± 0.29 (2.5 - 3.3)	3.4 ± 0.05 (3.4 - 3.5)

### Bioclimatic range modeling

**Figure 11.**
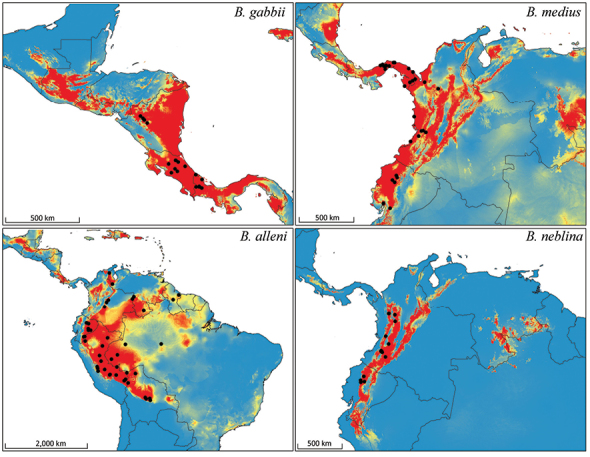
Bioclimatic distribution models and localities for *Bassaricyon* species. Models from MAXENT using all vouchered occurrence records, 19 bioclimatic variables, and one potential habitat variable.

**Figure 12.**
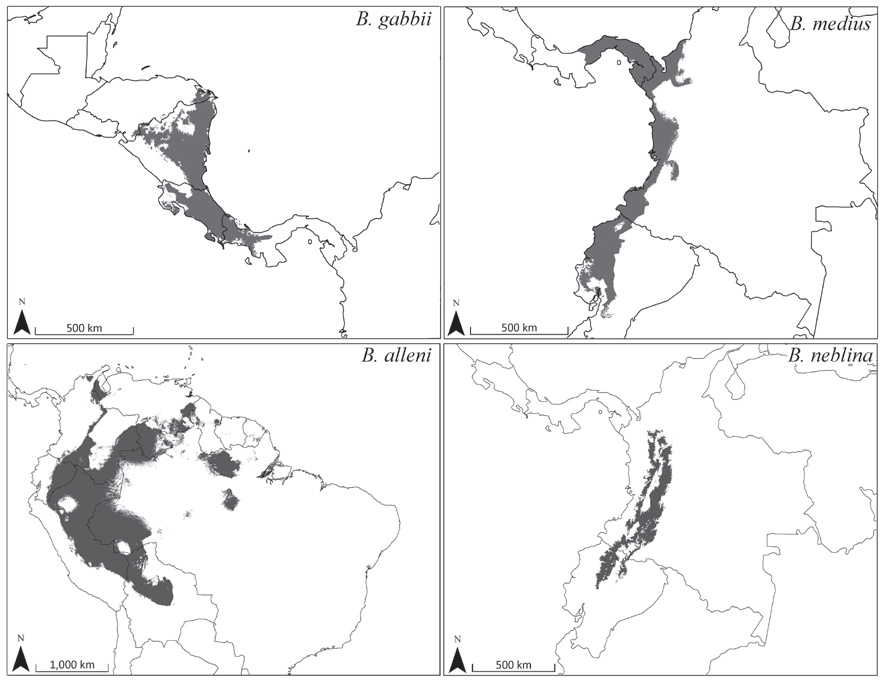
Predicted distribution for *Bassaricyon* species based on bioclimatic models. To create these binary maps we used the average minimum training presence for 10 test models as our cutoff. In addition, we excluded areas of high probability that were outside of the known range of the species if they were separated by unsuitable habitat.

Distribution models for all species are judged to have performed well based on their high values for ‘area under the curve’ (AUC) and unregularized test gain (Table 9), as well as their fit of the final prediction to the locality data (Figures 11–12). There was relatively low impact of withholding test data from these models, as indicated by the low Mean Test AUC values. These values are lowest for *Bassaricyon alleni*, probably reflecting its larger distribution relative to the variation of environmental data (Phillips et al. 2006). The strongest environmental predictors for *Bassaricyon neblina* sp. n. were seasonal variation in temperature (suitability declines with higher variation, after sharp threshold) and the temperature of the wettest quarter (negative relationship). The annual range of temperatures was the most important predictor for the *Bassaricyon gabbii* and *Bassaricyon medius* distributions (both sharp negative relationships). *Bassaricyon alleni* was the only one of the four species to have an ecological biome ranked as one of the top predictors (Tropical Moist Broadleaf Forests as highly suitable).

**Table 9.** Performance of bioclimatic distribution models for four *Bassaricyon* species using vouchered specimen localities. Mean values are averages of 10 models run, each withholding 20% of data as test localities, while the Full Model AUC used all available data. The mean value for equal training sensitivity and specificity was used as a logistic threshold to create a range map predicting presence/absence.

	Localities	Mean Test AUC (stdev)	Full Model AUC	Mean Unregularized Training Gain	Mean equal training sensitivity and specificity (logistic threshold)
*Bassaricyon alleni*	43	0.901 (0.036)	0.939	1.85	0.302
*Bassaricyon gabbii*	18	0.977 (0.012)	0.993	4.09	0.222
*Bassaricyon medius*	31	0.952 (0.028)	0.988	3.76	0.119
*Bassaricyon neblina*	16	0.996 (0.002)	0.998	4.77	0.160

The full Maxent distribution models predict the suitability of habitat across South and Central America (Figure 11). To make the binary prediction maps (Figure 12) we excluded areas with high probability that were disjunct from areas where specimens have been recorded (e.g., western Venezuela excluded from the map for *Bassaricyon neblina* sp. n., central and eastern Brazil excluded from the *Bassaricyon alleni* map, northern Central America excluded from the *Bassaricyon medius* map, South America excluded from the *Bassaricyon gabbii* map). For *Bassaricyon neblina* sp. n. we excluded areas of high probability from the Eastern Cordillera of Colombia and the Andes of southern Ecuador and northern Peru because of the lack of specimens. Likewise, predicted suitable habitat for *Bassaricyon gabbii* in northern Central America (Honduras, Guatemala) remains unverified by specimen data. Although there are two recent unconfirmed records in the region (Ordóñez Garza et al. 1999–2000), the specific locations of these sightings did not fall in areas predicted as suitable habitat by our models. Finally, the exact area of transition between *Bassaricyon gabbii* and *Bassaricyon medius* in Panama remains unclear. All of these regions should be considered high priority areas for future surveys, especially areas identified as potential *Bassaricyon neblina* sp. n. habitat (see Discussion, below).

The range of *Bassaricyon neblina* sp. n. is typical of many Andean species in being restricted to wet cloud forest habitats, which are limited in area and also under heavy development pressure. In comparing recent land use (Eva et al. 2004) of suitable historical *Bassaricyon neblina* habitat, we found that 42% of suitable habitats have been converted to agriculture or urban areas, and 21% remain in natural but largely unforested conditions. Thus we predict that only 37% (40,760 km^2^) of appropriate Olinguito habitats remain forested.

## Systematics

### Bassaricyon neblina sp. n.

http://zoobank.org/94DDB038-2111-44D1-A940-766BF8F15E51

http://species-id.net/wiki/Bassaricyon_neblina

#### Holotype.

We designate as the holotype of *neblina* specimen number 66753 in the mammalogy collection of the American Museum of Natural History, New York, a skin and complete skull of an old adult female, from Las Máquinas (= Las Machinas [see Voss 1988:474], *circa* 00°32’S, 78°39’W, 2130 m), Pichincha Province, Ecuador, collected 21 September 1923 by G.H.H. Tate.

#### Referred specimens.

**Figure 13.**
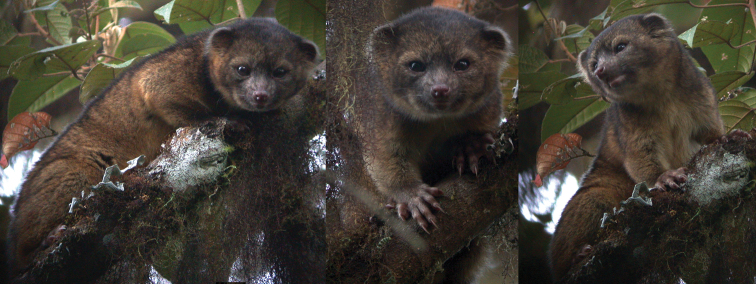
The Olinguito, *Bassaricyon neblina neblina*, in life, in the wild. Taken at Tandayapa Bird Lodge, Ecuador (for mammalogical background of Tandayapa, see Lee et al. 2006). Photograph by Mark Gurney.

QCAZ 0159, partial skin, Otonga Reserve, 1800 m, Cotopaxi Province, Ecuador; MECN 2177, adult female, skin and skull, La Cantera 2300 m, Cotopaxi Province, Ecuador; QCAZ 8661, young adult female, skin, skull, and postcranial skeleton, Otonga Reserve, 2100 m, Cotopaxi Province, Ecuador (collected by K. Helgen et al., August 2006); QCAZ 8662, young adult female, skin, skull, and postcranial skeleton, [“forested gully near”] La Cantera, 2260 m, Cotopaxi Province, Ecuador (collected by M. Pinto et al., August 2006). We have also seen photographs of this species from Tandayapa, 2350 m, Pichincha Province (Figure 13).

**Figure 14.**
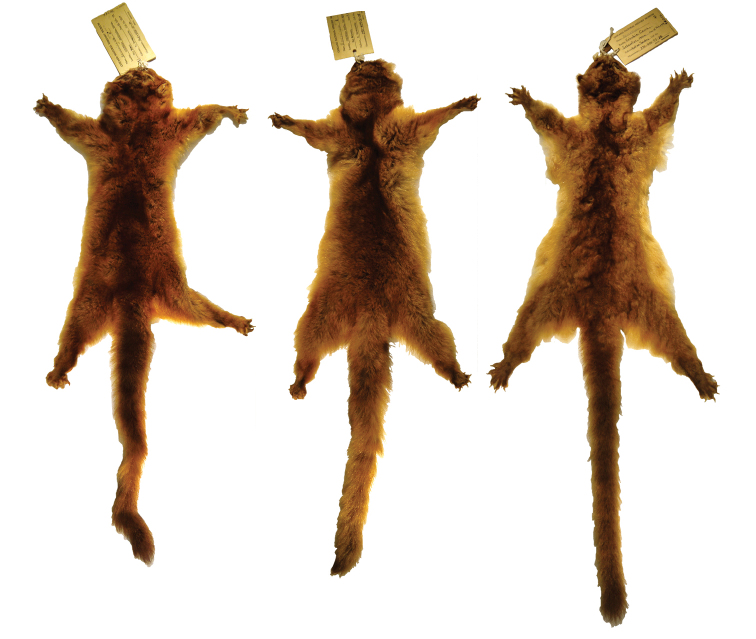
Olinguito skins from different regions of the Colombian Andes. Left, *Bassaricyon neblina ruber*, of the western slopes of the Western Andes of Colombia (FMNH 70722, adult male); Middle, *Bassaricyon neblina hershkovitzi*, of the eastern slopes of the Central Andes of Colombia (FMNH 70727, adult female); Right, *Bassaricyon neblina osborni*,of the eastern slopes of the Western Andes and eastern slopes of the Central Andes of Colombia (FMNH 90052, adult female).

**Figure 15.**
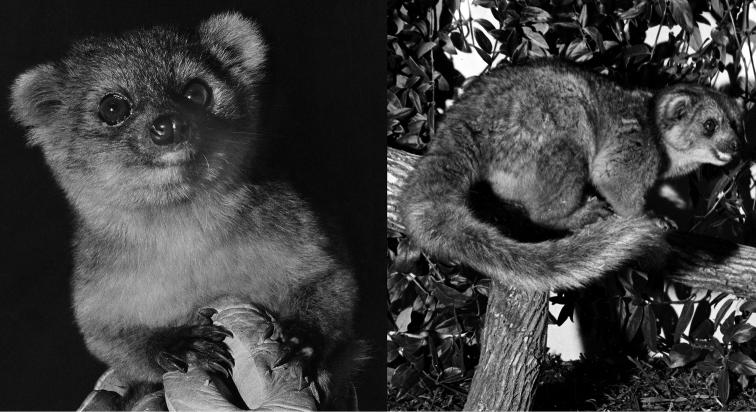
The Olinguito, *Bassaricyon neblina osborni*, in life. Photograph taken in captivity, at the Louisville Zoo (see Poglayen-Neuwall 1976). This animal, named “Ringerl”, was received as an adult in 1967 from the mountains of Colombia near Cali, and exhibited in various zoos, including the National Zoo in Washington, D.C. (see text). Photographs by I. Poglayen-Neuwall, previously unpublished (additional photographs published by Poglayen-Neuwall 1976).

**Figure 16.**
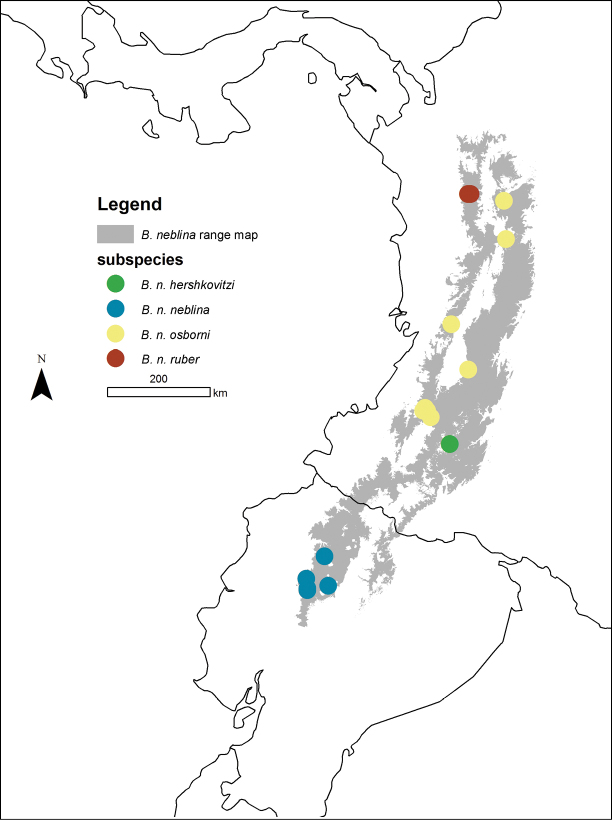
Distributions (localities) of the four Olinguito subspecies in the Andes of Colombia and Ecuador.

Below, we identify additional referred specimens when we describe three additional subspecies of *Bassaricyon neblina* from the cordilleras of Colombia (Figures 9–10, 13–16).

#### Diagnosis.

*Bassaricyon neblina* can be easily identified on the basis of both external and craniodental characteristics (Figures 3–7, Tables 3–5). It differs from other *Bassaricyon* in its smaller body and cranial size; longer, denser, and more richly coloured dorsal pelage (black-tipped, tan to strikingly orange- to reddish-brown); indistinctly banded, bushier, and proportionally shorter tail (at least compared to the lowland olingos, *Bassaricyon alleni* and *Bassaricyon medius*, Table 5); (externally) more rounded face with a blunter, less tapering muzzle; smaller and more heavily furred external ears, and considerably reduced auditory bullae, with a markedly smaller external auditory meatus; broadened and more elongate postdental palate (‘palatal shelf’), bearing more prominent lateral ‘flanges’ (sometimes developed to the point where it nearly closes off the “palatal notch” *sensu* Asher 2007); and proportionally much larger first molars (M1and m1), achieved especially by the development of more massive and bulbous principal molar cusps (protocone, paracone, metacone, hypocone) in M1, and for m1 by the widening of the talonid with the expansion in particular of the entoconid and hypoconid. The m1paraconid is reduced relative to other *Bassaricyon*.

Where *Bassaricyon medius* and *Bassaricyon neblina* occur in regional sympatry on the western slopes of the Andes, *Bassaricyon neblina* is smaller and more richly rufous and/or blackish in coloration, and is distinguished by all of the characteristics noted above. Externally, *Bassaricyon neblina* can only be confused with the highest elevation populations of *Bassaricyon alleni*, from forests above 1000 m on the eastern slopes of the Andes (specimens from Pozuzo and Chanchamayo in Peru), which, like *Bassaricyon neblina*, also have long, black-tipped dorsal pelage (though not so strongly rufous as in *Bassaricyon neblina*), ears that are especially furry (though not so small as in *Bassaricyon neblina*), and tails averaging slightly shorter than in lowland populations of *Bassaricyon alleni* (but not as short as in *Bassaricyon neblina*). The craniodental characteristics of *Bassaricyon neblina* (especially of the palate, bullae, and molars) are unmistakable.

#### Etymology.

The specific epithet *neblina* (Spanish, “fog or mist”), a noun in apposition, references the cloud forest habitat of the Olinguito.

#### Distribution.

The recorded distribution of *Bassaricyon neblina* comprises humid montane rainforests (“cloud forests”) from 1500 m to 2750 m in the Northern Andes, specifically along the western and eastern slopes of the Western Andes of Colombia and Ecuador, and along the western and eastern slopes of the Central Andes of Colombia (Figure 16). *Bassaricyon neblina* occurs in regional sympatry with *Bassaricyon medius medius* on the western slopes of the Ecuadorian Andes, where we have encountered the two species at localities less than 5 km apart. On the basis of our museum and field research, we document *Bassaricyon neblina* from 16 localities (representing 19 elevational records) in the Western Andes of Ecuador and the Western and Central Andes of Colombia. All sites are situated between 1500 and 2750 m (mean 2100 m, median 2130 m, ± 280 s.d.) and are associated with humid montane forest (“cloud forest”, Churchill et al. 1995). We used bioclimatic modeling to predict the global geographic distribution of *Bassaricyon neblina*, which comprises wet, forested ecoregions typical of the habitats where Olinguitos have been recorded (Figures 11–12). As noted above, of the entire land area predicted to be suitable for Olinguito occurrence, 42% has been converted to agriculture or urban areas and 21% comprises other unforested landscapes; only 37% (40,760 km^2^) of this land area is currently forested.

#### Geographic variation.

Geographic variation in the Olinguito is remarkable, reflecting consistent regional differences in color, size, and craniodental features associated with differential distributions in disjunct areas of the Andes. This is unsurprising given that the montane forests of the Central and Western Cordilleras of the Northern Andes are a region where major evolutionary differentiation has unfolded in many endemic Andean vertebrate groups (e.g., Benham 2012, Graham et al. 2010, Voss et al. 2002, Velasco et al. 2010). Below we diagnose four distinctive subspecies of *Bassaricyon neblina* and describe their geographic ranges as so far understood.

### Subspecies of *Bassaricyon neblina*

#### Bassaricyon neblina neblina subsp. n.

http://species-id.net/wiki/Bassaricyon_neblina_neblina

(western slopes of Western Andes of Ecuador)

##### Diagnosis.

This subspecies is (in skull length) smaller than *Bassaricyon neblina osborni* subsp. n., but larger than *Bassaricyon neblina hershkovitzi* subsp. n. and *Bassaricyon neblina ruber* subsp. n. (though *Bassaricyon neblina ruber* subsp. n. is more robust cranially, with a wider skull). It has proportionally very large teeth, especially P4 and the first molars, and a narrow skull, with a narrow and low-domed braincase (Figures 9–10, Table 8). In color it most closely resembles *Bassaricyon neblina osborni* subsp. n., but is the least rufous of the subspecies, usually with the greatest preponderance of black tipping to the fur (e.g., Figure 13).

##### Distribution.

The nominate subspecies is endemic to Ecuador, where it is recorded from the western slopes of the Andes, in Pichincha and Cotopaxi Provinces, in forests at elevations from 1800 to 2300 m (Figure 16).

##### Referred specimens.

As listed for *Bassaricyon neblina*, above.

#### Bassaricyon neblina osborni subsp. n.

http://species-id.net/wiki/Bassaricyon_neblina_osborni

(eastern slopes of Western Andes and western slopes of Central Andes of Colombia)

##### Diagnosis.

This is the largest subspecies of *Bassaricyon neblina*, with a short rostrum, widely splayed zygomata, wide rostrum and braincase, and very large molars and posterior premolars; the dorsal pelage is of moderate length, tan to orangish-brown in overall color, with prominent black and gold tipping, with a more grayish face and limbs, with the limbs bearing relatively short fur, and a tail usually grizzled with golden-brown fur tipping.

##### Distribution.

This is the representative of *Bassaricyon neblina* on the eastern slopes of the Western Andes of Colombia (e.g., Castilla Mountains [AMNH]; Sabanetas [FMNH]; El Tambo [NMS]; the vicinity of Cali [Poglayen-Neuwall 1976]; El Duende [Saavedra-Rodríguez and Velandia-Perilla 2011]; Gallera: “western slope of most eastern ridge of southern Western Andes” [AMNH, Paynter 1997:222]) and the western slopes of the Central Andes of Colombia (Cerro Munchique [FMNH]). One specimen (AMNH 42351, from Santa Elena, Antioquia Department) derives from the eastern slopes of the Central Andes in northern Colombia (habitat described as “deforested, grassy, and bushy (Chapman 1917:61)”; Paynter 1997:403); this shows that this subspecies also crosses to the eastern slopes of the Central Andes in Antioquia. Further south, in the department of Huila, the smaller subspecies *Bassaricyon neblina hershkovitzi* subsp. n. (see below) occurs on the eastern slopes of the Central Andes.

Records to date of *Bassaricyon neblina osborni* are from 1500 to at least 2750 m elevation in Cauca, Valle del Cauca, and Antioquia Departments of Colombia (Figure 16). *Bassaricyon medius medius* is also recorded from the Cauca Valley (east slopes of Western Andes and western slopes of Central Andes) at elevations up to at least 725 m (UV-3774: Saavedra-Rodríguez and Velandia-Perilla 2011; see account of *Bassaricyon medius* below), so these two taxa (*Bassaricyon medius medius* and *Bassaricyon neblina osborni*) are presumably regionally sympatric (and probably elevationally stratified) across the range of this Olinguito subspecies on the slopes of the Western and Central Andes.

##### Etymology.

The name honors Henry Fairfield Osborn (1857–1935), paleontologist, faculty of Princeton and Columbia Universities, and Curator of Vertebrate Paleontology (1891–1909) and President (1909–1933) of the American Museum of Natural History (Gregory 1937, Colbert 1996). “*Bassaricyon osborni*” is a manuscript name (never formally published) associated with a specimen of this taxon (AMNH 32609, with “Type” written on the skull), demonstrating a century-old intention, later discarded (probably by J.A. Allen or H.E. Anthony, see below), to name this taxon after Osborn. Here we validate this unpublished name as a newly described subspecies of *Bassaricyon neblina*, but we choose a more complete specimen than AMNH 32609, which has a damaged mandible and various broken teeth, as holotype.

##### Holotype.

FMNH 88476, adult male, skin and skull, Munchique, 2000 m, Cauca Department, Colombia (collected by K. von Sneidern, 3 June 1957).

##### Paratypes.

AMNH 32608, adult female, skin and skull, and AMNH 32609, adult male, skin and skull, Gallera (Chapman 1912:155; = “La Gallera” of Paynter 1997:222), 5000 feet (=1524 m), Cauca Department, Colombia (both collected by L. Miller, 13 July 1911); NMS A59-5083, adult female, skin and skull, El Tambo, 1700 m, Cauca Department, Colombia (collected by K. von Sneidern); FMNH 85818, adult male, skin and skull, Munchique, 2000 m, Cauca Department, Colombia (collected by K. von Sneidern, 19 January 1956); FMNH 89220, adult female, skin and skull, Sabanetas, 2000 m, Cauca Department, Colombia (collected by K. von Sneidern, 26 September 1957); FMNH 90052, adult female, skin and skull, Sabanetas, 1900 m, Cauca Department, Colombia (collected by K. von Sneidern, 12 February 1959).

##### Referred specimens.

AMNH 14185, skin (skull not found), adult male, Castilla Mountains (“La Castilla” of Paynter 1997), Valle del Cauca Department (collected by J.H. Batty, 9 June 1898); AMNH 42351, adult male, skin and skull, Santa Elena, apparently at 9000 feet (= 2750 m), Antioquia Department, Colombia (collected by H. Niceforo Maria, 10 January 1919) (Paynter 1997:403); USNM 598996, adult male, skin, skull, and postcranial skeleton, from Colombia, specific locality unknown (received from Tulane University).

#### Bassaricyon neblina hershkovitzi subsp. n.

http://species-id.net/wiki/Bassaricyon_neblina_hershkovitzi

(eastern slopes of Central Andes of Colombia)

##### Diagnosis.

This is the smallest subspecies of *Bassaricyon neblina*, with the fur of the dorsum and tail very long, and richly orange-brown (brown with strong golden and black tipping) in coloration, and more golden brown face and limbs, with the limbs well-furred. The skull, braincase, and rostrum are especially narrowed, the posterior palatal shelf is extremely broad, and the molars are proportionally very large.

##### Distribution.

This is the representative of *Bassaricyon neblina* on the eastern slopes of the Central Andes of southern Colombia (Figure 16). Records to date are from 2300 to 2400 m elevation in the vicinity of San Antonio (Huila Department), a forested locality “on eastern slope of Central Andes at headwaters of Rio Magdalena, near San Agustin” (Paynter 1997:380) (see Kattan et al. 1994).

##### Etymology.

The name honors American mammalogist Philip Hershkovitz (1909–1997), collector of the type series, Curator of Mammals at the Field Museum of Natural History (1947–1974; Emeritus Curator until 1997), and authority on South American mammals (Patterson 1987, 1997).

##### Holotype.

FMNH 70727, adult female, skin, skull, and postcranial skeleton, San Antonio, 2300 m, San Agustin, Huila Department, Colombia (collected by P. Hershkovitz, 6 September 1951) (see Figure 18).

##### Paratypes.

FMNH 70724, adult male, skin, skull, and postcranial skeleton, San Antonio, 2400 m, San Agustin, Huila Department, Colombia (collected by P. Hershkovitz, 20 August 1951); FMNH 70725, adult male, skin, skull, and postcranial skeleton, San Antonio, 2400 m, San Agustin, Huila Department, Colombia (collected by P. Hershkovitz, 25 August 1951); FMNH 70726, adult male, skin, skull, and postcranial skeleton, San Antonio, 2300 m, San Agustin, Huila Department, Colombia (collected by P. Hershkovitz, 6 September 1951).

#### Bassaricyon neblina ruber subsp. n.

http://species-id.net/wiki/Bassaricyon_neblina_ruber

(Urrao District, western slope of Western Andes of Colombia)

##### Diagnosis.

This subspecies is markedly smaller (at least in skull length) than *Bassaricyon neblina neblina* and *Bassaricyon neblina osborni*, with the fur longest and most strikingly reddish of all the Olinguito populations (reddish with golden and black tipping), and more golden brown face and and reddish brown limbs, with the limbs well-furred. Though similar in overall skull length to *Bassaricyon neblina hershkovitzi*, the skull is especially wide for its size (Table 8), with broad zygomata, braincase, and rostrum compared to that subspecies.

##### Distribution.

This subspecies is recorded from the Urrao District of Colombia (2200–2400 m in Huila and Antioquia Departments), on the western slope of the Western Andes, where it is documented by specimens collected in 1951 by Philip Hershkovitz.

##### Etymology.

The name refers to the rich reddish-brown pelage of this subspecies (Figures 3, 14).

##### Holotype.

FMNH 70722, adult male, skin, skull, and postcranial skeleton, Rio Urrao, 2400 m, Urrao, Huila Department, Colombia (collected by P. Hershkovitz, 24 April 1951).

##### Paratypes.

FMNH 70721, adult female, skin, skull, and postcranial skeleton, Rio Ana, 2200 m, Urrao, Huila Department, Colombia (collected by P. Hershkovitz, 19 April 1951); FMNH 70723, adult male, skin, skull, and postcranial skeleton, Guapantal, 2200 m, Urrao, Antioquia Department, Colombia (collected by P. Hershkovitz, 28 April 1951).

### Reproductive isolation and genetic divergence of *Bassaricyon neblina*

Information from sympatric occurrences and captive breeding demonstrates that the Olinguito, *Bassaricyon neblina*, is reproductively isolated from other species of *Bassaricyon* and clearly constitutes a distinct “biological species” (i.e., *sensu* Mayr 1940, 1942).

**Figure 17.**
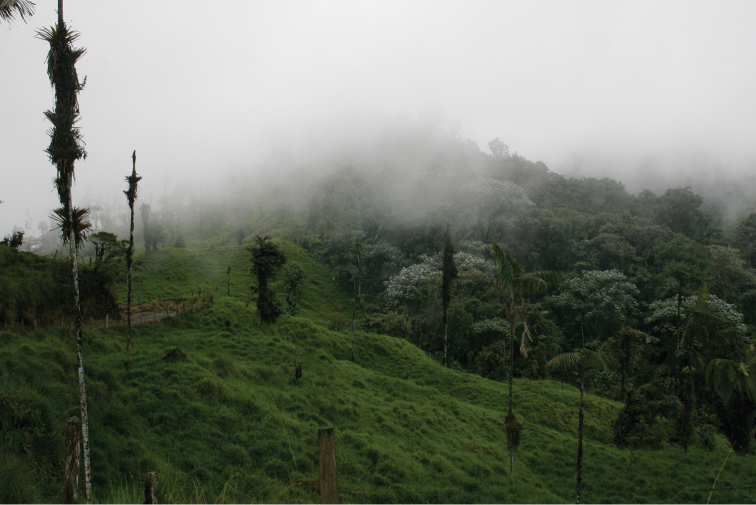
Area of sympatric occurrence between *Bassaricyon* species in western Ecuador. Farmland cutting into cloud forest habitat at Las Pampas, approximately 1800 m, on the western slopes of the Western Andes, Ecuador, along the boundary of Otonga, a protected forest reserve. It is at this elevational and environmental boundary that *Bassaricyon medius medius* (lower elevations, including more anthropogenically disturbed habitats) and *Bassaricyon neblina neblina* (higher elevations, less disturbed forests) co-occur in regional sympatry on the western slopes of the Andes.

In Ecuador we documented the Olinguito (*Bassaricyon neblina neblina*) in regional sympatry with the Western lowland olingo, *Bassaricyon medius medius*; we recorded the two species at localities less than 5 km apart (i.e., at Otonga and San Francisco de las Pampas) during fieldwork in August 2006. The ecogeographic relationship between the two species is probably one of elevational parapatry or limited elevational overlap along the western slopes of the Andes. *Bassaricyon medius medius* extends into the elevational range of *Bassaricyon neblina*,perhaps especially in areas where cloud forests have been cleared for human settlement, agriculture, and pastoralism (Figure 17).

**Figure 18.**
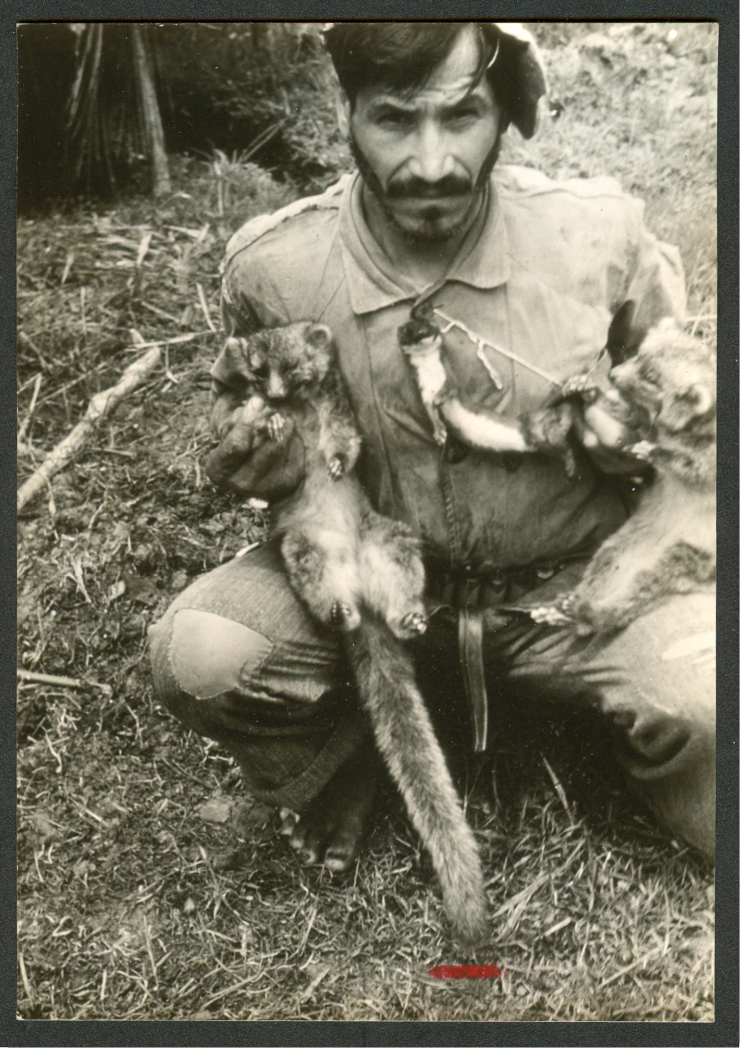
Type series of an Olinguito subspecies, *Bassaricyon neblina hershkovitzi*, in the field. Two Olinguito specimens (FMNH 70726, paratype of *hershkovitzi*, and FMNH 70727, holotype of *hershkovitzi*, along with a Long-tailed weasel, *Mustela frenata*, FMNH 70998) brought in by a local hunter, 6 September 1951, at San Antonio, San Agustín, Huila District, Colombia. Photo by P. Hershkovitz, courtesy of the Field Museum of Natural History.

Ingeborg Poglayen-Neuwall (pers comm. to R. Kays, 2006) informed us that an adult female zoo animal named “Ringerl” (Figure 15; also figured by Poglayen-Neuwall 1976), which we can now identify as an Olinguito (*Bassaricyon neblina osborni*), was moved among several zoos during the 1970s because it would not successfully breed with other captive olingos (i.e., not *Bassaricyon neblina*), most of which were apparently *Bassaricyon alleni* (see Poglayen-Neuwall 1976).

The Olinguito differs from congeners (*Bassaricyon alleni*, *Bassaricyon medius*, and *Bassaricyon gabbii*) by 9.6–11.3% in base-pair composition of the (mitochondrial) cytochrome *b* gene (Table 2), a level of divergence consistent with that separating biological species in many groups of mammals, including carnivores (Baker and Bradley 2006). For comparison with other procyonids, this level of genetic distinction is equivalent to the 10–11% divergence between *Procyon lotor* and *Procyon cancrivorus*, sympatrically-occurring raccoons traditionally classified in separate subgenera (Goldman 1950, Helgen and Wilson 2005), and comparable to the 9–13% divergence between *Nasua narica* and *Nasuella olivacea* (Helgen et al. 2009), coatis traditionally classified in separate genera (Hollister 1915, Decker and Wozencraft 1991, Wozencraft 1993, 2005).

**Karyotype:** The karyotype of an adult female Olinguito(*Bassaricyon neblina osborni*, then identified as “*Bassaricyon gabbii*”, with 2n = 38, as in all procyonids) was reported and discussed (but not described in detail) by Wurster-Hill and Gray (1975), and figured by Nash (2006). This was based on a captive animal originally captured from mountains in the vicinity of Cali in Colombia (Figure 15).

**Description:** The Olinguito is the smallest species of *Bassaricyon*, both in skull and body size (Tables 3, 5), and is thus, on average, the smallest living procyonid (matched only by small individuals of the Ringtail, *Bassariscus astutus*). The tail averages 10% longer than the head-body length (Table 5). The pinnae are proportionally much smaller in *Bassaricyon neblina* than in other *Bassaricyon*, appearing shorter and rounder, and standing out less conspicuously on the head; they are also more heavily furred and usually fringed with a paler, contrasting border of buffy or golden fur. The dorsal fur is dense, long, and luxurious, with the longer hairs measuring 30–40 mm in length (usually much shorter in other *Bassaricyon*, at least in the predominantly lowland taxa *Bassaricyon medius* and *Bassaricyon alleni*, but reaching 25 mm in the highest-elevation populations of *Bassaricyon alleni* on the eastern versant of the Andes). The hairs of the dorsum, crown, upper limbs, and tail are golden-orange, with grey bases and dark red-brown or blackish-brown tips, generating a distinctly dark, often red-brown appearance, more striking than the relatively drab fur colors (more tan or yellowish-brown to grayish-brown) of other *Bassaricyon* (Figure 3). The fur of the cheeks, chin, venter, and underside of the limbs is yellow to the bases, often washed with orange. The fur of the face in front of the eyes is shorter and gray or buff with black tipping, sometimes with a pale cream ring around the eyes. The hairs of the tail are strongly tipped with gold, or with both golden and blackish-brown tipping. In contrast to specimens of other *Bassaricyon*, the tail is not conspicuously banded, though when viewed in the right light, a banding pattern of alternating golden and brown hues is weakly apparent in some specimens. A white terminal tail tip is present in a minority of individuals.

Like other *Bassaricyon*, the cranium of *Bassaricyon neblina* is long relative to its width, with a moderately long and broad rostrum, an elongate and somewhat globose braincase with a smooth dorsal surface, and moderately developed postorbital processes.

**Figure 19.**
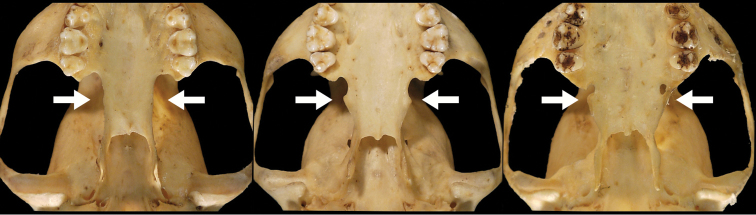
Lateral flare of the postpalatal shelf in *Bassaricyon*. Lateral extension of the postpalatal shelf (shown by white arrows) is usually absent or little-developed in other *Bassaricyon* (e.g., left, *Bassaricyon alleni*, FMNH 41501), but is well-developed in *Bassaricyon neblina* (e.g., center, *Bassaricyon neblina ruber*, FMNH 70721, and right, *Bassaricyon neblina hershkovitzi*, FMNH 70726).

In *Bassaricyon neblina*, the temporal ridges do not meet to form a sagittal crest, even in older animals. The postdental palate is usually flared laterally, but is smoothly parallel-sided, tapers posteriorly, or bears only weaker bony flaring in other *Bassaricyon* (Figures 4–7, 19). At its more extreme development (e.g., in FMNH 70726), the portion of the bony palate sitting behind M2 is almost continuously joined to the postdental palate by a continuous shelf of bone, rather than bearing a deep excavation separating the molar-bearing portion of the bony palate from the postdental shelf (Figure 19). The auditory bullae are very small in the Olinguito relative to other *Bassaricyon*, both in length and vertical inflation, and the external auditory meatus is considerably narrower in diameter, on average (Figures 4–7). The median septal foramen of the anterior palate (Steno’s Foramen), between the paired incisive (or anterior palatal) foramina, is usually well-developed. The mandible is proportionally less elongate than in other *Bassaricyon*, with a proportionally larger and more vertically-oriented coronoid process (Figures 4–5). The first two upper premolars are caniniform, similar in size and shape to those of other *Bassaricyon*. P3 is usually relatively smaller in *Bassaricyon neblina* than in other *Bassaricyon*. P4 is similar in structure to congenersbut is relatively larger with a more bulbous protocone and more prominent metacone. M1 and M2 are proportionally lengthened and considerably more massive in appearance, especially relative to skull size, than in other *Bassaricyon*. p4 is variable in size among *Bassaricyon neblina* subspecies, generally smaller than other *Bassaricyon* in *Bassaricyon neblina ruber* and *Bassaricyon neblina hershkovitzi*, but proportionally quite large in *Bassaricyon neblina neblina*. m1 is relatively much larger in *Bassaricyon neblina* than in other *Bassaricyon*;each of the four major cusps that define the subrectangular shape of this tooth are massive and bulbous, and the posterior portion is especially broadened, with the metaconid and hypoconid particularly large and laterally expanded relative to congeners. m2 is also often expanded in size in *Bassaricyon neblina* relative to other *Bassaricyon*.

**Natural history:** Our field observations document that *Bassaricyon neblina* is nocturnal, arboreal, frugivorous, and probably largely solitary (compiled during July and August 2006 at Otonga Forest Reserve in Ecuador: 00°41’S, 79°00’W; for faunal and floral context see Freiberg and Freiberg 2000, Nieder and Barthlott 2001, Jarrín-V 2001). It occupies cloud forest canopies and is an adept leaper. It has a single pair of mammae and probably raises one young at a time. Notes associated with AMNH 14185, the first specimen to arrive in a museum, mention that it was “shot at 2 pm [an error for 2 am?] in high trees while coming down mountain to feed on guavas; strictly nocturnal.”

An adult female Olinguito (an animal named “Ringerl”, *Bassaricyon neblina osborni*, Figure 15) that lived at the Louisville Zoological Park and the National Zoological Park in Washington during 1967–1974 made vocalizations different from those of other *Bassaricyon* according to Poglayen-Neuwall (1976). Poglayen-Neuwall (1976) figured a picture of this animal in characteristic estrus behavior and in various other circumstances (see below for more discussion of this captive Olinguito).

**Previous identifications and references**:

Though described taxonomically for the first time in this paper, the Olinguito (heretofore misidentified as other species of *Bassaricyon*) has been represented in museum collections for more than a century, has been exhibited in zoos, has had its karyotype published, and has been included in published molecular phylogenetic studies.

Olinguito museum specimens previously reported in the literature include specimens from Gallera, Colombia, mentioned by Allen (1912, 1916) (AMNH 32608 and 32609, as “*Bassaricyon medius*”); a specimen from Santa Elena, Colombia, reported by Anthony (1923) (AMNH 42351, as “*Bassaricyon medius*”), specimens from “El Duende Regional Reserve” (2200 m asl; 04°02'55.6"N, 76°27'28.4"W)” and “Los Alpes, Florida, 2250 m asl” in Valle del Cauca Department, Colombia (mammal collection of the Universidad del Valle, Cali, Colombia, specimen numbers 12736, 13700) discussed by Saavedra-Rodríguez and Velandia-Perilla (2011) (as “*Bassaricyon gabbii*”); and a skull from San Antonio, Huila Department, Colombia (FMNH 70727) figured by Prange and Prange (2009) (as “*Bassaricyon gabbii*”, designated above as the holotype of *Bassaricyon neblina hershkovitzi*). One Olinguito specimen, AMNH 32609, bears an unpublished scientific name, *“Bassaricyon osborni*”, written on the skull and on the tags, apparently during the early twentieth century—correctly reflecting an understanding that the specimen represented an undescribed species. This appellation (a “manuscript name”) is likely attributable to J.A. Allen or H.E. Anthony (seemingly too early to be G.H.H. Tate). In any case, the name was never published, and by 1923, Allen had passed away (in 1921) and Anthony had decided that the specimen in question was best referable to *Bassaricyon medius* (see Anthony 1923). We have chosen to validate this name under our own authorship, above, as a subspecies of *Bassaricyon neblina*.

Mejía Correa (2009) reported camera-trap photos of a species of *Bassaricyon* at Munchique in Colombia; these records presumably represent *Bassaricyon neblina osborni*, the only *Bassaricyon* recorded at Munchique.

Molecular data for *Bassaricyon neblina* from a cell line were first generated and used in a phylogenetic study of carnivore relationships by Ledje and Arnason (1996a, 1996b), apparently the same animal whose karyotype was reported and discussed by Wurster-Hill and Gray (1975) (also Nash 2006). DNA sequence data (*12S* rRNA, cytochrome *b*) from this sample, available on Genbank, have been used in various other published studies (e.g., Flynn and Nedbal 1998, Koepfli and Wayne 1998, Emerson et al. 1999, Flynn et al. 2000, Gaubert et al. 2004, Marmi et al. 2004, Flynn et al. 2005, Fulton and Strobeck 2007, Yonezawa et al. 2007, Wolsan and Sato 2010, Nyakatura and Bininda-Emonds 2012, but not in some important studies, e.g., Koepfli et al. 2007, Agnarsson et al. 2010). This cell line apparently originated from the zoo animal “Ringerl” (discussed by Poglayen-Neuwall [1976]), an adult female Olinguito (*Bassaricyon neblina osborni*, originally from mountains near Cali, Colombia), apparently exhibited at the Louisville Zoo, National Zoo, Tucson Zoo, Bronx Zoo, and possibly Salt Lake City Zoo during the late 1960s and 1970s (Ingeborg Poglayen-Neuwall, pers. comm. to R. Kays, 2006). Ivo Poglayen-Neuwall (in litt. to C.O. Handley, Jr., 6 November 1964) mentioned another *Bassaricyon*, a young adult male at the Louisville Zoo, also from Cali, received in 1964, that seems also to have been an Olinguito (“shows the following unusual physical features: (1) strikingly round head… (2) very short, round ears! (3) rather short tail (no amputation!)”). This latter animal seems not to be discussed in Poglayen-Neuwall’s various publications on olingos, and it is unclear what became of it.

### Bassaricyon gabbii

J. A. Allen, 1876:23.

http://species-id.net/wiki/Bassaricyon_gabbii

Northern Olingo

Bassaricyon richardsoni J.A. Allen, 1908:662.
Bassaricyon lasius Harris, 1932:3.
Bassaricyon pauli Enders, 1936:365.

#### Type specimens and localities.

The holotype of *gabbii* is USNM A14214, an unsexed adult skull (with dimensions, in this sexually dimorphic species, that indicate that the specimen is female). The holotype skull, collected by William Gabb, was figured by Allen (1908). No specific locality other than Costa Rica was given in the original description (Allen 1876), but Allen (1877) later specified its origin as “Talamanca” (i.e., the Talamanca Mountains of southeastern Costa Rica; see also Allen 1908:667). The skin associated with the specimen was lost before the species was described (Allen 1876). Allen (1877) later incorrectly associated the skin of a coati, *Nasua narica*, with the holotype skull, but corrected this mistake soon after (Allen 1879).

The holotype of *richardsoni* is AMNH 28486, an adult female (skin and skull), from “Rio Grande (altitude below 1,000 feet), Atlantic Slope”, Nicaragua (Allen 1908). The skull of the type was figured by Allen (1908).

The holotype of *lasius* is UMMZ 64103, an adult male (skin and skull), from “Estrella de Cartago… six to eight miles south of Cartago near the source of the Rio Estrella, … about 4500 feet”, Costa Rica (Harris 1932).

The holotype of *pauli* is ANSP 17911, an adult male (skin and skull), from “between Rio Chiriqui Viejo and Rio Colorado, on a hill known locally as Cerro Pando, elevation 4800 feet, about ten miles from El Volcan, Province de Chiriqui”, Panama (Enders 1936).

#### Diagnosis.

**Figure 20.**
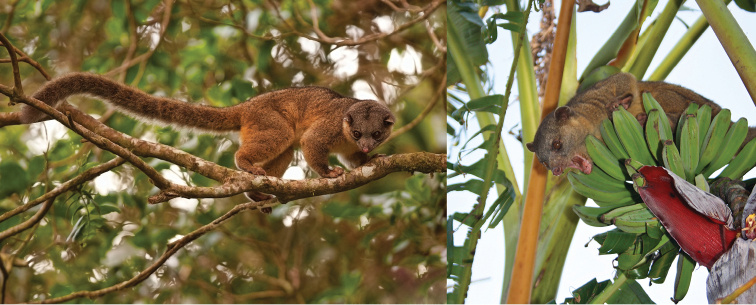
Northern Olingo, *Bassaricyon gabbii*, in life, in the wild. Photographed at Monteverde, Costa Rica by Greg Basco (left) and Samantha Burke (right).

This is the largest olingo, measuring larger than all other taxa in most measurements, and is the most sexually dimorphic species of *Bassaricyon* in cranial characters and measurements (Table 3). It can be distinguished externally from other olingo species by its coloration, which is grayish-brown (less rufous than in other *Bassaricyon*), with the face usually dominated by gray, the belly fur cream-colored (sometimes washed with orange), and the tail showing a faint banding pattern (Figures 3, 20). Fur length on the dorsum varies noticeably with elevation (longer at higher elevation). The tail is similar in length to the head-body length, averaging shorter relative to total length than in other olingos (Table 5), perhaps an indication of less complete arboreal habits than in other *Bassaricyon* (an aspect unfortunately not captured well in our Figure 3).

The skull is large compared to other *Bassaricyon* (Table 3), with the zygomata more widely splayed, particularly in males (Figures 4–5, Table 3), and a wide rostrum. Uniquely in *Bassaricyon*, a sagittal crest develops in older males. The cheekteeth and auditory bullae are proportionally quite small compared to the size of the skull, relatively smaller than in *Bassaricyon medius* and *Bassaricyon alleni*, and the postpalatal shelf tends to be broadened relative to *Bassaricyon medius* and *Bassaricyon alleni*. The canines are more massive than in other *Bassaricyon*. The first lower molar (m1) is distinctively shaped relative to other *Bassaricyon*, with the paraconid usually situated right at the midpoint of the front of the tooth and often jutting out anteriorly (the m1 paraconid is less prominent and/or situated more antero-medially in other *Bassaricyon*).

#### Distribution.

This species occurs in the central portion of Central America, in montane and foothill forests, from northern Nicaragua to Costa Rica and into the Chiriqui Mountains in western Panama, possibly also extending north into Honduras and Guatemala (Reid 2009; see below). Northern olingos are recorded at elevations as low as sea level, but are most commonly encountered in forests above 1000 m, and extend elevationally at least as high as 1700 m (USNM 324293), and probably as high as the upper limit of forest on the highest peaks in Costa Rica. Forested areas above 1000 m in Central America can be understood to be the core distribution of this species. Vouchered records are from the north-central mountains of Nicaragua (Allen 1908, AMNH, USNM); the mountains of Costa Rica, including the slopes stretching down to the Atlantic coast (Allen 1877, Allen 1908, Harris 1932, Goodwin 1946, Wilson 1983, Timm et al. 1989, Reid 1997, Timm and LaVal 2000, de la Rosa and Nocke 2000, Wainwright 2007, Reid 2009) and a few records of observations from the Pacific slopes (Puntarenas Province: Daily et al. 2003; Guanacaste Province: González-Maya and Belant 2010); and in the Chiriqui Mountains of western Panama (Enders 1936, ANSP, USNM). Reid (2009) included the Azuero Peninsula of Panama in a distribution map for *Bassaricyon*, but we can trace no record from this region and the basis of this record is unclear (F. Reid, R. Samudio, J. Pino, *in litt.*, 2012–2013). The eastern limits of occurrence for this species are not yet firmly established, but the boundary of occurrence between *Bassaricyon gabbii* and *Bassaricyon medius orinomus* apparently lies between 81 and 80 degrees longitude in central Panama. Ours is the first study to document the marked taxonomic distinction between *Bassaricyon gabbii* of (especially montane) central Mesoamerica, including western Panama, and *Bassaricyon medius orinomus* of eastern Panama, the Central American member of a group of closely related lowland taxa that also includes *Bassaricyon medius medius* (of northern South America west of the Andes) and *Bassaricyon alleni* (of South America east of the Andes). The nature of the interactions between *Bassaricyon gabbii* and *Bassaricyon medius orinomus* at this boundary (whether involving, e.g., parapatry, sympatric overlap, or limited hybridization) is unknown and a priority for field study (see Figures 11–12).

There are unverified records of olingos occurring north of Nicaragua, in Honduras and Guatemala, and these records may represent *Bassaricyon gabbii*. Ordóñez Garza et al. (1999–2000) reported a night sighting of an olingo in Honduras at “La Picucha, Montaña de Babilonia, 1380 m, Parque Nacional Sierra de Agalta, Departamento de Olancho” and discussed a museum specimen of an olingo (later apparently lost) obtained from hunters in Guatemala near the Honduras border at “Montaña Cerro Negro Norte… Río Bobos… 300–500 m” in the Sierra del Merendón, Departamento de Izabal” (Ordóñez Garza et al. 1999–2000, McCarthy and Pérez 2006). Neither of these localities is immediately adjacent to large contiguous areas of *Bassaricyon gabbii* occurrence as predicted by our range modeling analyses (Figure 11), but both areas could represent relevant habitats for the Northern Olingo, and verifying the occurrence of olingo populations in Honduras or Guatemala should be considered an important goal in Mesoamerican mammalogy.

#### Geographic variation.

The nominal taxa *richardsoni*, *lasius*, and *pauli*, synonymized here with *Bassaricyon gabbii*, were all originally diagnosed based on distinctions in pelage coloration and fur length, in small samples (Allen 1908, Harris 1932, Enders 1936), and their taxonomic status has never been closely reviewed.

Specimens from northern Nicaragua have fur that is slightly more suffused with orange tones than animals from Costa Rica or western Panama. Nicaraguan populations may deserve recognition as a distinct subspecies, *Bassaricyon gabbii richardsoni*, as sometimes classified (e.g., Goodwin 1946, Hall 1981), but specimens from Nicaragua are too rare in collections for a detailed assessment, and Nicaraguan animals are otherwise very similar to specimens from Costa Rica and Panama. A young adult female specimen of *Bassaricyon gabbii* from Almirante in Panama’s Bocas del Toro Province (USNM 316320) is one of few low-elevation records for *Bassaricyon gabbii*, and is notable in having much smaller teeth than specimens from higher-elevation forests in the adjacent Chiriqui Mountains, and deserves close study in the future. We have carefully examined the type series of the nominal taxa *Bassaricyon lasius* and *Bassaricyon pauli*, the morphological attributes of which clearly fall into the range of variation seen in series we refer to *Bassaricyon gabbii*. We confidently relegate these names, often previously recognized as distinct species known only from the type localities (e.g., Hall 1981, Nowak 1999, Wozencraft 2005), to the synonymy of *Bassaricyon gabbii*, although we note that the only specimen of *gabbii* that we have seen from the area of the type locality, the Talamanca Range, is the holotype, which is an adult with worn teeth and no accompanying skin. Without further investigation, ideally involving the compilation and study of greater number of museum specimens from throughout the range of this species, we do not yet advocate recognizing subspecies of *Bassaricyon gabbii*, though we note that names are available for several of the major sections of the Middle American Highlands (Cordillera de Talamanca: *gabbii*, Chiriqui: *pauli*, Cordillera Central: *lasius*, Nicaraguan Highlands: *richardsoni*).

#### Notes.

This is the olingo speciesmost commonly seen and photographed by visitors to the Neotropics, especially because it is present at Monteverde and several other protected areas in Costa Rica that are frequently visited by both tourists and biologists (e.g., Forsyth 2008, Kays 2009, Reid 2009). It is larger, more sexually dimorphic, and has a shorter tail than other olingo species, suggesting a different ecology and behavior compared to the slightly better studied *Bassaricyon medius* and *Bassaricyon alleni* (see accounts below). *Bassaricyon gabbii* has been reported feeding on fruit and nectar in rainforest trees, but no details have been published on its diet. Olingos in Monteverde, Costa Rica, are often seen during the day, typically as solitary animals; it is unclear if diurnal activity is typical for the species or if this is in response to being fed by humans at the tourist lodge (Reid 2009, Kays 2009). Relevant field notes associated with *Bassaricyon gabbii* include: “fruit in stomach” (ANSP 18851); “shot in fruit tree at night” (ANSP 18852); “lactating” on 4 June 1937 (ANSP 18894); “shot at 8:00 pm in small trees” (ANSP 17911); mother with accompanying young on 20 August 1909 (AMNH 30748).

#### Specimens examined.

**Costa Rica**: AMNH 140334, LACM 26480, 64837, UMMZ 64103 (holotype of *lasius*), 112321, 112322, USNM A14214 (holotype of *gabbii*). **Nicaragua**: AMNH 28486 (holotype of *richardsoni*), 30748, 30749, USNM 337632, 338859. **Panama**: AMNH 147772, ANSP 17911 (holotype of *pauli*), 18850, 18851, 18852, 18893, 18894, BMNH 3.12.6.3, 5.5.4.5, KU 165554, MCZ 38506, TCWC 12941, USNM 316230, 324293, 324294, 516945, 516946.

### Bassaricyon alleni

Thomas, 1880:397.

http://species-id.net/wiki/Bassaricyon_alleni

Eastern Lowland Olingo

Bassaricyon beddardi Pocock, 1921a: 231.
Bassaricyon medius siccatus Thomas, 1927:80.

#### Type specimens and localities.

The holotype of *alleni* is BMNH 80.5.6.37, an adult female (skin and skull), from “Sarayacu, on the Bobonasa River, Upper Pastasa River …this must not be confused with the far larger and better known Sarayacu on the Ucayali in Peru”, in Amazonian Ecuador (Thomas 1880). An image of the holotype as a living animal was figured by Thomas (1880: plate XXXVIII), and the anatomy of this specimen was further discussed by Mivart (1885, 1886).

The holotype of *beddardi* was an adult female, from “Bastrica Woods, Essequibo River”, Guyana (Flower 1895; Pocock 1921a), and was an animal that lived in the London Zoological Gardens from 1894 to 1900 (Beddard 1900; Allen 1908). Aspects of the internal anatomy of this specimen were described in detail by Beddard (1900), and the skull of this specimen was figured and discussed by Pocock (1921a), who also mentioned the specimen was prepared as a skeleton (Pocock 1921b), but apparently this specimen was not retained as a museum specimen in the collections of the BMNH, and now cannot be found (D. Hills, BMNH, *in litt.*, 2004).

The holotype of *siccatus* is BMNH 27.5.3.2, an adult female (skin and skull), from “Guaicaramo, on the Llanos of Villavicencio, east of Bogata, about 1800 feet”, Colombia (Thomas 1927).

#### Diagnosis.

**Figure 21.**
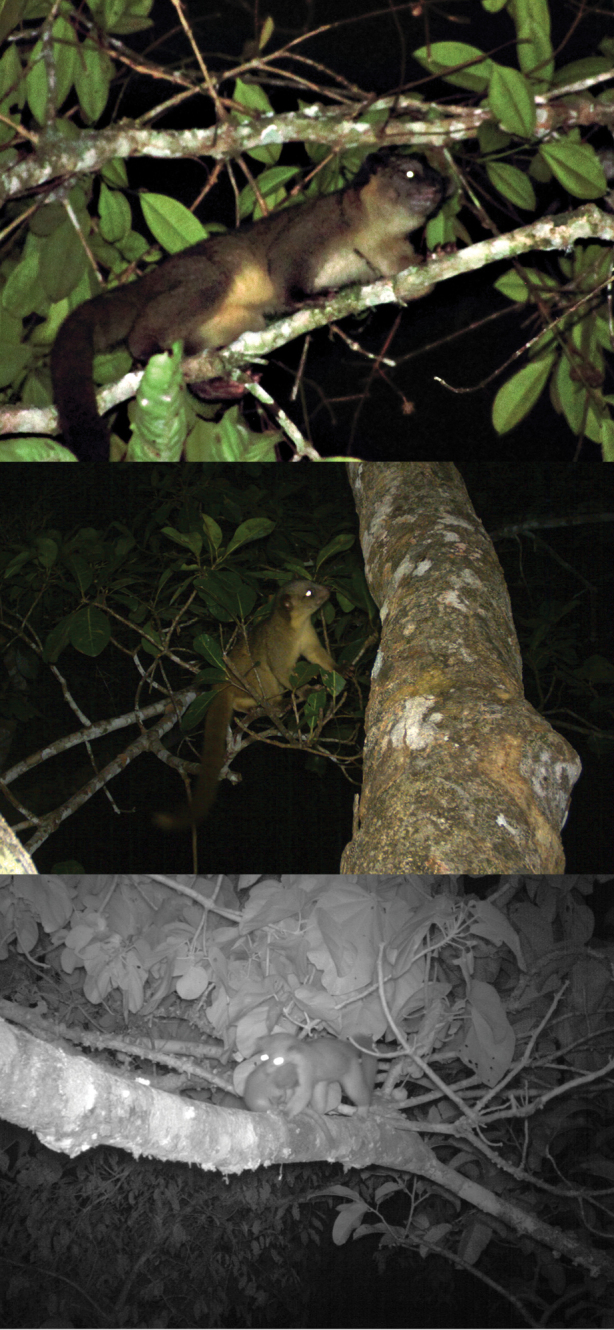
Eastern Lowland Olingo, *Bassaricyon alleni*, in life, in the wild. Top, photographed at night (accentuating the dark tones in the pelage) at La Esperanza (Distrito de Yambrasbamba, Provincia de Bongará, Departamento Amazonas), 2000 m, northern Peru; Middle, color camera trap photo in forest canopy, from confluence of the Camisea and Urubamba Rivers (11°42'S, 72°48'W). Peru; Bottom, infrared camera trap photo in forest canopy (same locality as middle photo), showing an olingo carrying a baby in its mouth. Top photograph by César M. Aguilar; middle and bottom camera trap photos courtesy of Smithsonian Conservation Biology Institute.

**Figure 22.**
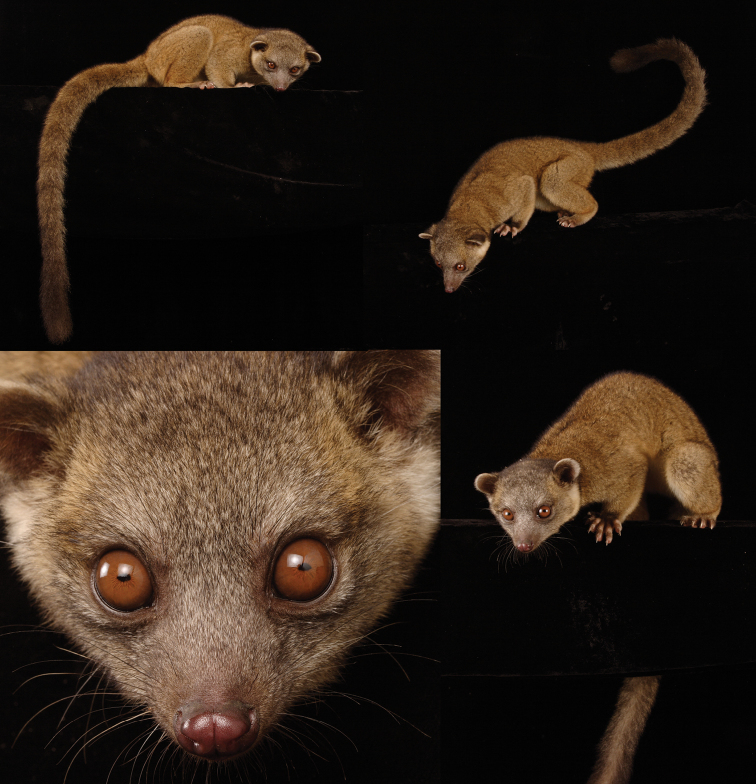
Western Lowland Olingo, *Bassaricyon medius medius*, in life. A wild animal photographed under studio conditions at Las Pampas, adjacent to Otonga Reserve, Ecuador. Photographs courtesy of P. Asimbaya and L. Velásquez.

*Bassaricyon alleni* is a medium-sized olingo, smaller than *Bassaricyon gabbii* of Mesoamerica, and larger than *Bassaricyon neblina* of the Andes. It requires closest comparison with the closely-related and allopatrically-distributed taxon *Bassaricyon medius*, from which it differs especially in having (externally) more strikingly black-tipped dorsal pelage (giving the pelage a slightly darker appearance in *Bassaricyon alleni*), (cranially) in its proportionally wider and (on average) shorter rostrum, and in having more inflated auditory bullae (Table 3), and (dentally) in its generally larger p4 (Table 4). *Bassaricyon alleni* is considerably larger than *Bassaricyon medius medius* (of South America west of the Andes), such that there is a clear body size contrast between the two lowland olingo taxa of South America (*Bassaricyon alleni* east of Andes *vs. B. medius medius* west of Andes), but is very similar in size to *Bassaricyon medius orinomus* (of eastern Panama). *Bassaricyon medius orinomus* often has a reddish tail that contrasts somewhat with the less rufous head and body; *Bassaricyon alleni* tends to be more uniformly colored head to tail. In life, *Bassaricyon alleni* usually has a darkly pigmented nose, whereas in *Bassaricyon medius* the nose is often pink (Ivo Poglayen-Neuwall to C.O. Handley Jr., *in litt.*, 9 February 1973; Figures 21–22). Sequence divergence in cytochrome *b* in these sister species (*Bassaricyon alleni*, *Bassaricyon medius*), separated by the Andes, is 6–7% (Table 2).

#### Distribution.

This is the only species of *Bassaricyon* found east of the Andes. *Bassaricyon alleni* has a wide distribution in forests on the eastern slopes of the Andes and in lowland forests east of the Andes, with records from forested areas of Venezuela (Thomas 1920, Handley 1976, Bisbal 1989, 1993, Ochoa et al. 1993, Linares 1998, BMNH, USNM), Guyana (Pocock 1921a, Lim and Engstrom 2005, ROM), eastern Colombia (Thomas 1927, Donegan and Salaman 1999, AMNH, BMNH, USNM), eastern Ecuador (Thomas 1880, 1920, Schulenberg and Aubrey 1997, Pitman et al. 2002, Tirira 2007, Borman et al. 2007, Alverson et al. 2008, Pinto and Tirira 2011b, BMNH, EPN, FMNH, MCZ, QCAZ), eastern Peru (Thomas 1920, Grimwood 1969, Patton et al. 1982, Terborgh et al. 1984, Aquino and Encarnación 1986, Janson and Emmons 1990, Woodman et al. 1991, Pacheco et al. 1993, Pitman et al. 2003, 2004, Emmons et al. 1994a, 1994b, Emmons and Romo 1994, Boddicker 1997, Emmons 2001, Rodríguez and Amanzo 2001, Emmons et al. 2001, Vriesendorp et al. 2004, Alverson et al. 2008, Gilmore et al. 2010, BMNH, FMNH, MVZ, UMMZ, USNM, ZMB), northwestern Bolivia (Crespo 1959, Emmons 1991, Redford and Stearman 1993, Anderson 1997, Alverson et al. 2000, 2003, Alverson 2003, Ríos-Uzeda and Arispe 2010), and western Brazil (Calouro 1999, Kays and Russell 2001, Vaz 2004, Oliveira 2009, Magalhães-Pinto et al. 2009, Sampaio et al. 2010).

In Guyana, *Bassaricyon alleni* is recorded only from two specimens, the type of *beddardi* (Pocock 1921a, see above) and a specimen from Iwokrama Forest (Lim and Engstrom 2005, at ROM); there are no records to date from either Suriname or French Guiana, where it might be expected to occur (Tate 1939, Husson 1978, Voss et al. 2001, Lim et al. 2005).

In Brazil, the only firm records are from southwestern Amazonia (the states of Amazonas and Acre) (Calouro 1999, Kays and Russell 2001, Vaz 2004, Oliveira 2009, Magalhães-Pinto et al. 2009, Sampaio et al. 2010), though it is likely to occur also in Roraima and Pará (Figures 11–12). Brazilian Amazonian records of olingos from the state of Roraima, as “*Bassaricyon beddardi*” (Mendes Pontes and Chivers 2002, Mendes Pontes et al. 2002, Mendes Pontes 2004, 2009, Cheida et al. 2006), are thus far apparently based on misidentifications of kinkajous, *Potos* (Sampaio et al. 2011).

The elevational range of *Bassaricyon alleni* as documented by museum specimens extends from sea level to 2000 m. The great majority of records originate from lowland forests below 1000 m, but specimens from Ecuador and Peru (especially from Chanchamayo) have been collected from 1100 to 2000 m (specimens at BMNH, FMNH, USNM). It seems likely that the distribution of *Bassaricyon alleni* extends higher on the eastern slopes of the Andes than that of *Bassaricyon medius* does on the western slopes because of the apparent absence of *Bassaricyon neblina* on the eastern versant of the Andes.

#### Karyotype.

The karyotype of a male *Bassaricyon alleni* (2n = 38, NF = 68; then identified as “*Bassaricyon gabbii*”) was reported and described by Wurster and Benirschke (1967, 1968) based on an animal at the National Zoo (Washington, D.C.)—most likely USNM 395837, an adult male received from Leticia, Amazonas District, Colombia (the only male olingo at the zoo at the time).

#### Geographic variation.

Some geographic variation is apparent in *Bassaricyon alleni*, and several taxonomic names have been applied to different regional representatives of this species, including in the western Amazon (typical *alleni* Thomas 1880), Guyana (*beddardi* Pocock 1921a), and the Eastern Andes of Colombia (*siccatus* Thomas 1927).

The most notable morphological distinction that we have observed within *Bassaricyon alleni* is between lowland specimens (from forests below 1000 m) and specimens collected in montane forests above 1000 m in the Eastern Andes (e.g., Chanchamayo and Pozuzo in Peru). Specimens from these higher elevations have somewhat shorter tails and are more brownish (less orange tones in pelage), with notably longer fur, and greater development of black tipping to the fur, though the pelage is not as long and luxurious as in *Bassaricyon neblina*. Press reports of a possibly new species of *Bassaricyon* discovered in the Tabaconas – Namballe National Sanctuary in the Eastern Andes of Peru (e.g., Hance 2012), where *Bassaricyon alleni* is predicted to occur (Figure 11–12), may refer to such a highland population of *Bassaricyon alleni*.

*Bassaricyon beddardi* of Guyana has often been recognized as a species distinct from *Bassaricyon alleni* in checklists and inventories (e.g., Lim and Engstrom 2005, Reid and Helgen 2008c, Sampaio et al. 2011, Wozencraft 1993, 2005), but supporting justification has been lacking. The holotype of *beddardi*, originally a zoo animal, appears to be lost (see above). However, both the holotype (as described by Beddard [1900] and Pocock [1921a]) and a second (and the only additional) specimen from Guyana (ROM 107380, from Iwokrama Forest) closely correspond in their morphological characteristics to Amazonian and Andean specimens of *Bassaricyon alleni*, and our molecular comparisons demonstrate little molecular divergence between the ROM specimen and a specimen of *Bassaricyon alleni* from the Peruvian Amazon (Table 1; 1.3% sequence divergence in cytochrome *b*), such that we suggest that *Bassaricyon beddardi* can be regarded as a synonym of *Bassaricyon alleni*. We allocate the name *siccatus* to the synonymy of *Bassaricyon alleni* based on geography and craniodental morphology of the type specimen, but further, more detailed study of geographic variation across the range of *Bassaricyon alleni* would be welcome, perhaps focused in particular on variation across different regions of the Eastern Andes (cf. Thomas 1920, 1927). At present, we recognize no subspecies within *Bassaricyon alleni*.

#### Notes.

Though this is the most widely distributed member of the genus (Figure 12), relatively little is known of this species in the wild. Brief notes about the ecology and behavior of wild *Bassaricyon alleni* are included in the publications of Aquino and Encarnación (1986), Emmons (1990, 1991), Janson and Emmons (1990), and Patton et al. (1982). However, captive olingos described and discussed in detail by Poglayen-Neuwall and Poglayen-Neuwall (1965) (also Poglayen-Neuwall 1966, 1989) were all (or almost all) *Bassaricyon alleni*, originally from the vicinity of Iquitos (Amazonian Peru), such that for behavior under captive conditions, *Bassaricyon alleni* is the best studied member of the genus. Most olingos discussed by Poglayen-Neuwall (1976) were probably also *Bassaricyon alleni*, though one animal, an adult female named “Ringerl” (Figure 15), was an Olinguito, *Bassaricyon neblina osborni* (see account of *Bassaricyon neblina*, above). Poglayen-Neuwall’s (1973) delightful popular article, “The Odorous Olingo,” remains one of the most concentrated sources of firsthand information for this species (and olingos in general), discussing how *Bassaricyon alleni* is highly arboreal but will cross open spaces on the ground, is active from sunset to dawn, is predominantly frugivorous but also eats some animal matter (small rodents and lizards, nestling birds, insects, and eggs), has little social organization beyond mother-offspring pairs, displays a high intensity of scent marking in both sexes, flees and releases a foul-smelling odor when threatened, has one young following a 72–74 day gestation period, and that males are aggressive with one another and cannot be housed together. Relevant (and limited) field notes associated with *Bassaricyon alleni* include: “stomach contents fruits and a green vegetable pulp” (USNM 194315); “lactating” on 7 April 1967 (USNM 443717).

#### Specimens examined.

**Colombia**: AMNH 70532, 142223, BMNH 27.5.3.2 (type of *siccatus*), USNM 281482, 281483, 281484, 281485, 395837, 544415. **Ecuador**: AMNH 67706, BMNH 14.4.25.38, 80.5.6.37 (holotype of *alleni*), EPN “4”, RM0151, FMNH 41501, 41502, MCZ 37920, 37921, 37922, 37923, QCAZ 3371, YPM 1458, 1459. **Guyana**: ROM 107380. **Peru**: AMNH 98653, 98654, 98662, 98709, BMNH 5.11.2.6, 27.1.1.70, 1912.1.15.3, 1922.1.1.17, FMNH 34717, 65787, 65789, 65805, 86908, 86909, 98709, MVZ 153646, 155219, 155220, UMMZ 107907, USNM 194315, 194316, 255121, 255122, ZMB 63197. **Venezuela**: BMNH 99.9.11.25, USNM 443279, 443717, 443718.

### Bassaricyon medius

Thomas, 1909

http://species-id.net/wiki/Bassaricyon_medius

Western Lowland Olingo

Bassariscyon [sic] *gabbi* [sic] *orinomus* Goldman, 1912:16.

#### Type specimens and localities.

The holotype of *medius* is BMNH 9.7.17.10, an adult male (skin and skull) from “Jimenez, mountains inland of Chocó, W. Colombia, 2400 feet” (Thomas 1909).

The holotype of *orinomus* is USNM 179157, an adult male (skin and skull), from “Cana (altitude 1,800 feet), in the mountains of eastern Panama” (Goldman 1912). Goldman (1912) figured the holotype skull.

#### Diagnosis.

*Bassaricyon medius* is a medium-sized olingo, smaller (on average) than *Bassaricyon gabbii* of Mesoamerica and larger than *Bassaricyon neblina* of the Andes. It requires closest comparison with the closely-related, allopatrically-distributed taxon *Bassaricyon alleni*, from which it differs especially in having (externally) less strikingly black-tipped dorsal pelage (which gives the pelage a slightly darker appearance in *Bassaricyon alleni*), (cranially) in its proportionally narrower and (on average) longer rostrum, and in having less inflated auditory bullae (Table 3), and (dentally) in its generally smaller p4 (Table 4). *Bassaricyon medius medius* is considerably smaller than *Bassaricyon alleni* (of South America east of the Andes), such that there is a clear body-size contrast between the two lowland olingo taxa of South America (*Bassaricyon alleni* east of Andes *vs. B. medius medius* west of Andes), but *Bassaricyon medius orinomus* (of eastern Panama and northwestern Colombia) is very similar in size to *Bassaricyon alleni*. *Bassaricyon medius orinomus* often has a reddish tail that contrasts somewhat with the less rufous head and body; *Bassaricyon alleni* tends to be more uniformly colored head to tail. In life, *Bassaricyon alleni* usually has a darkly pigmented nose, whereas in *Bassaricyon medius* the nose is often pink (Ivo Poglayen-Neuwall to C.O. Handley Jr., *in litt.*, 9 February 1973; Figures 21–22). Sequence divergence in cytochrome *b* in these sister species (*Bassaricyon medius*, *Bassaricyon alleni*), separated by the Andes, is 6–7% (Table 2).

#### Distribution and geographic variation.

*Bassaricyon medius* occurs in forests from Central Panama to Colombia and Ecuador west of the Andes, where it is recorded from sea level up to about 1800 m elevation. We recognize two distinctive subspecies of *Bassaricyon medius*, distinguished especially by clear differences in size (Tables 6–7).

*Bassaricyon medius medius* (Figure 22) occurs in most of the South American portion of the range, where it is recorded west of the Andes in western Colombia (Thomas 1909) and western Ecuador (Lönnberg 1921, Parker and Carr 1992, Tirira 2008, Pinto and Tirira 2011a), in the Chocó region, on the western slopes of the Andes, and in outlying western ranges. It occurs in regional sympatry with *Bassaricyon neblina* at Otonga–San Francisco de las Pampas and probably elsewhere along the western versant of the Andes.

**Figure 23.**
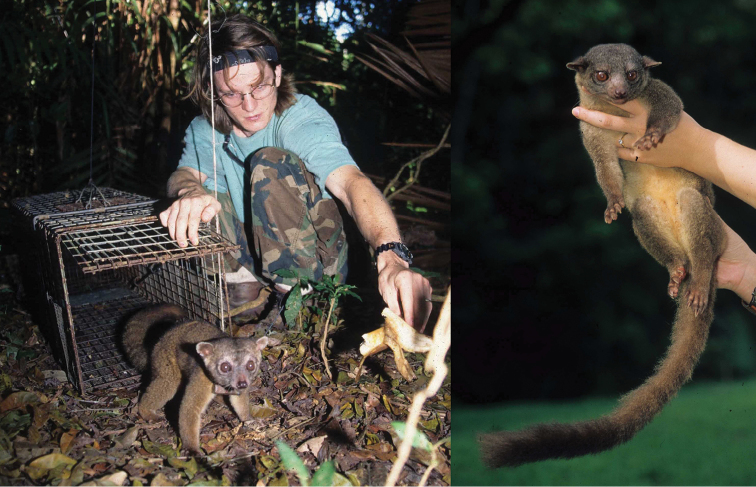
Western Lowland Olingo, *Bassaricyon medius orinomus*, in life. Wild animals captured, radio-collared, released, and studied by Roland Kays in Limbo Plot, Pipeline Road, Gamboa, Panama (Kays 2000). Photographs courtesy of M. Guerra and R. Kays.

*Bassaricyon medius orinomus* (Figure 23) occurs primarily in the Central American portion of the range, where it is recorded from central and eastern Panama, from the vicinity of the Canal Zone in the west to the Darien Mountains in the east (Goldman 1912, 1920, Handley 1966, Mendez 1970, Kays 2000). In the Darien, records include Cerro Tacarcuna and Cerro Pirri (USNM series), and as these mountain blocks extend across the Colombian border, there can be no doubt this subspecies enters South America in northwestern Colombia. The two north-westernmost records of *Bassaricyon medius* in Colombia, from Villa Arteaga, Antioquia District (FMNH 69578) and from the Cauca Valley (AMNH 37797) have large anterior premolars relative to *Bassaricyon medius medius* and we tentatively attribute them to *Bassaricyon medius orinomus*, with *Bassaricyon medius medius* then recorded further south in forests of the Chocó and Western Andean slopes. The specimen from the Cauca Valley (from Puerta Valdivia, Antioquia District: Allen 1916) demonstrates that *Bassaricyon medius* also occurs in lowland forests in between the Western and Central Andes; another low elevation specimen from the Cauca Valley of Colombia reported by Saavedra-Rodríguez and Velandia-Perilla (2011) (UV-3774, Río Agua Sucia, Río Cajambre, 725 m) that we have not examined is also presumably *Bassaricyon medius*.

As noted above for *Bassaricyon gabbii*, the nature of interactions with the distribution of *Bassaricyon gabbii* on the western margin of the range of *Bassaricyon medius* in Panama (whether characterized by allopatry, parapatry, or sympatry) is unknown and worthy of study.

#### Notes.

The first detailed description of representatives of this species was published in French by Huet (1883), based on two specimens from Chorrera, near Panama City (Thomas 1909, Goldman 1920), the first specimens to become available after the original discovery of *Bassaricyon gabbii* in Costa Rica in 1876 (Allen 1876). Thomas (1909) was first to name it (*Bassaricyon medius*), based on specimens from western Colombia, and Goldman (1912, 1920) described the larger Panamanian subspecies (*orinomus*) and provided brief notes on the species, noting its arboreal, nocturnal, and frugivorous habits, and its association in fruiting trees with *Potos*. More recent field studies in Panama (Kays 2000, Kays et al. 2012, Figure 23) are limited but likewise show *Bassaricyon medius* to be nocturnal, arboreal, frugivorous (and nectarivorous), and to feed in the same trees as kinkajous, which sometimes displace the smaller olingos. It is mostly solitary at night, spends its days in tree holes or other arboreal hide-outs, and usually has one young at a time. One olingo was found to have a home range of 37 hectares. Field workers have described this species’ vocalizations as “whey-chuck”, “wer-toll”, or “wake-up.”

Relevant field notes associated with *Bassaricyon medius* include: “shot at dusk in high tree in forest” (FMNH 29180); “shot at 8 pm, 40 feet up in large tree, active and agile, but curious, eyes shine brightly” (USNM 305748); “shot at 8:30 pm in avocado plantation” (USNM 305749); “shot near banana plantation (at night), stomach with banana” (USNM 305750); ” “shot at 8:30 pm in large tree in cafetal [coffee plantation], stomach with soft fruit with tomato-like seed” (USNM 305751); “shot at 8 pm in forest” (USNM 307037); “lactating” and pregnant with “1 embryo”, “stomach: fruit pulp” (USNM 310666); “shot in tree at night” (USNM 335767, 338348); “shot at night in tree in forest” (USNM 335769); “shot at night in tree in cocoa grove” (USNM 335770); “shot in small tree in plantain patch at night” (USNM 335771); “one embryo” in a pregnant female “shot in forest” (USNM 363342); “shot in banana tree” (USNM 363343)

#### Specimens examined.

***Bassaricyon medius medius***

**Colombia**: BMNH 9.7.17.10 (holotype of *medius*), 9.7.17.11, FMNH 29180, 86852, 90049, 90051, MVZ 124112, USNM 598997. **Ecuador**: AMNH 66752, BMNH 34.9.10.81, 34.9.10.82, EPN 841, 900, MECN DAP37, NMS A59-5081, A59-5082, QCAZ 8758, 8659.

***Bassaricyon medius orinomus***

**Panama**: USNM 171138, 179053, 179157 (holotype of *orinomus*), 179158, 179779, 179917, 206123, 284773, 284903, 284933, 284934, 284935, 305748, 305749, 305750, 305751, 305752, 305753, 305754, 307035, 307036, 307037, 310666, 310667, 310668, 324295, 324296, 335767, 335768, 335769, 335770, 335771, 338348, 338894, 363342, 363343, 363344. **Colombia** (tentatively attributed): AMNH 37797, FMNH 69578.

## Discussion

### Carnivore taxonomy

Descriptions of new species of carnivores are especially rare, and the order Carnivora is generally considered one of the most completely characterized groups across the entire tree of life (Collen et al. 2004, Reeder et al. 2007). *Bassaricyon neblina* is a deeply divergent lineage within its genus, a very morphologically distinctive member of the family Procyonidae, and even shows signs of evolutionary diversification across its geographic range. It thus adds significantly to current understanding of taxonomic, phylogenetic, and ecomorphological evolution in the family Procyonidae. It has presumably been overlooked by taxonomists for several reasons—principally the lack of close taxonomic attention paid to Neotropical procyonids for nearly a century (Helgen and Wilson 2003, Helgen et al. 2009), but probably also because of its nocturnal and arboreal habits, relatively limited geographic distribution, and the small number of specimens scattered across various museum collections (see Patterson 1994, 2000).

The description of the Olinguito highlights how incompletely known the taxonomy of almost all kinds of mammals remains, including the Carnivora (Gutiérrez and Helgen 2013). Our study of olingo taxonomy is part of a series of studies that have better clarified species diversity in insufficiently studied genera of Carnivora, especially in Neotropical small carnivores (e.g., *Procyon*: Helgen and Wilson 2002, 2003, 2005; Helgen et al. 2008b; *Nasuella*: Helgen et al. 2009; *Galictis*: Bornholdt et al. 2013), but also in other little-known genera (*Arctonyx*: Helgen et al. 2008a; *Eupleres*: Goodman and Helgen 2010), often revealing considerable overlooked biodiversity in poorly studied groups. Many additional carnivore genera have not been the subject of modern integrative systematic reviews, especially in the Neotropics (e.g., *Potos*, *Nasua*, *Conepatus*). Detailed reviews of these groups are likely to reveal additional overlooked diversity.

### Conservation

The rapid and ongoing discovery of endemic mammals and birds in northern Andean cloud forests (e.g., Robbins and Stiles 1999, Anderson and Jarrín-V 2002, Cuervo et al. 2001, 2005, Lara et al. 2012, Ojala-Barbour et al. in press) reaffirms the evolutionary importance of these unique habitats and betrays how incompletely inventoried this biota remains. Though a center of diversity and endemism for many groups (e.g., Young et al. 2002, Brehm et al. 2005, Mittermeier et al. 2005, Hughes and Eastwood 2006, Patterson et al. 2012), northern Andean cloud forests are among the most threatened ecosystems in the Neotropics (Young 1994, Myers et al. 2000, Mittermeier et al. 2005, Schipper et al. 2008). Drawing on the criteria used by the International Union for the Conservation of Nature (IUCN; Schipper et al. 2008; in this case, based on inferred population declines due to habitat declines over last three generations), we suggest classifying the Olinguito under the IUCN category of “Near Threatened.” Given that Olinguitos are directly dependent on cloud forest for habitat and food, deforestation appears to be the primary threat to Olinguito populations, and this IUCN categorization reflects our concerns about habitat destruction across its relatively restricted geographic range. Based on our distribution model, it appears that 42% of potential Olinguito habitat in Colombia and Ecuador has already been converted to agriculture or urban environments. Remaining habitat is highly fragmented and faces increasing threats from farming, grazing, deforestation for drug cultivation, logging, and climate change (Kattan et al. 1994, Myers et al. 2000, Brooks et al. 2002, Sarmiento 2002, Armenteras et al. 2003). The long-term survival of *Bassaricyon neblina* will depend on the preservation of those upland forest fragments that remain, and restoration of degraded habitat to maintain connectivity between populations. Its discovery introduces a novel flagship species around which to rally conservation initiatives in the region. Preserving cloud forests in this region would benefit the long-term conservation of the Olinguito, and many other Northern Andean cloud forest endemics.

Based on their relatively expansive distributional ranges, all of which include various protected areas (Figures 11, 12), we suggest IUCN Red List rankings of “Least Concern” for *Bassaricyon alleni*, *Bassaricyon medius*, and *Bassaricyon gabbii*, for the present.

### Biogeography

A well-resolved taxonomy for olingos has never been available, such that biogeographic patterns within the genus, and their origins, have never before been critically reviewed (Eizirik 2012). Our overview of *Bassaricyon* allows us to glimpse these patterns for the first time, unveiling both anticipated and unexpected biogeographic patterns.

Previous overviews of procyonid biogeography have focused especially on the important potential role of the Great American Biotic Interchange (GABI) in the diversification of the family (Marshall et al. 1979, Koepfli et al. 2007, Eizirik 2012). We complement this focus by suggesting that northern Andean uplift, proceeding in greatest part since the middle Miocene (Gregory-Wodzicki et al. 2000, Ollier 2006, Weir 2006), has played an almost equally important role in procyonid diversification.

The most detailed previous phylogenetic comparisons of olingos (Koepfli et al. 2007) highlighted the genetic divergence between taxa originating from North America and from South America (*Bassaricyon medius* from Panama [then called “*Bassaricyon gabbii*” by Koepfli et al. 2007] and *Bassaricyon alleni* from Peru), finding that this split apparently postdated the GABI. This comparison was undertaken prior to the discovery of the Olinguito lineage, the deepest split in the genus, and could not resolve the question of whether the radiation of crown group olingos unfolded first in North or in South America. Our phylogenetic comparisons indicate that *Bassaricyon neblina*, an Andean cloud forest endemic, is the sister taxon to all other *Bassaricyon* and last shared a common ancestor with congeners3–4 million years ago, a timescale concordant with the timing of both the GABI and Northern Andean mountain-building. That *Bassaricyon* mainly occurs in South America, with only one species, *Bassaricyon gabbii*, endemic to Central America, and that the earliest divergence in *Bassaricyon* is between *Bassaricyon neblina* and the other three species allows us to suggest that the most important events in the diversification of crown group *Bassaricyon* occurred in northwestern South America (as suggested by Poglayen-Neuwall 1973) (see Velazco and Patterson [2013] for particularly clear example of this same biogeographic pattern). That the two earliest divergences within the genus involve what are today montane (*Bassaricyon neblina* of the Andes) or mostly montane (*Bassaricyon gabbii* of the Costa Rican, Nicaraguan, and western Panama highlands) taxa provides an indication that the isolation of upland Neotropical habitats was likely important in the early diversification of the genus. Uplift of the Andes simultaneously created a barrier to dispersal that is ultimately reflected in the speciation event between the allopatric pair of lowland olingos, *Bassaricyon alleni* (eastern, cis-Andean) and *Bassaricyon medius* (western, trans-Andean) (cis- and trans-Andean *sensu* Haffer 1967). In addition to promoting evolutionary diversification within *Bassaricyon*, Northern Andean uplift has fostered the evolution of other endemic montane procyonids (the Mountain coatis *Nasuella olivacea* and *Nasuella meridensis* [Helgen et al. 2009] as well ascurrently unrecognized montane species of *Nasua*, synonymized uncritically with *Nasua nasua* under current taxonomic checklists [following Decker 1991]). These mountains also served as a key barrier to dispersal of presumed recent North American procyonid immigrants (*Procyon lotor* and *Nasua narica*) to South America, which penetrate South America only west of the Andes, primarily in western Colombia (Marín et al. 2012), with *Nasua narica* perhaps extending also to western Ecuador (Decker 1991) and *Procyon lotor* perhaps also to western Venezuela (Helgen and Wilson 2005).

The phylogenetic topology seen in *Bassaricyon*, with an Andean species sister to a clade of lowland congeners, is unusual among mammals, but seen in some groups with lowland representatives restricted to the Amazon. For example, the echimyid rodent genera *Dactylomys* and *Isothrix* present this pattern, with *Isothrix barbarabrownae* and *Dactylomys peruanus* restricted to the Andes and their congeners to the Amazon lowlands (Patterson and Velazco 2008, Lim 2012, Patterson et al. 2012). In olingos, the time estimates for this diversification are broadly equivalent with the estimated Pliocene divergence timing (2–5 mya) proposed between *Isothrix barbarabrownae* and its lowland congeners (Upham and Patterson 2012). A similar pattern of inferred colonization from the Andes to the Amazonian lowlands was proposed for dendrobatid frogs, but occurred earlier, during the late Miocene (11.2 – 5.3 mya), when the Andes were considerably lower in elevation (Santos et al. 2009).

One species of olingo, *Bassaricyon alleni*,is endemic to habitats east of the Andes, especially the Amazon. The Amazon is arguably the most diverse region of the planet (e.g. Bass et al. 2010, Malhado et al. 2013; but see Solari et al. 2012), and it has been postulated that its high current diversity is a result of an accumulation of lineages for a prolonged period of time, covering mostly the Pliocene and Miocene, with subsequent local divergences (e.g., Hoorn et al. 2010; Leite and Rogers 2013). However, *Bassaricyon alleni* appears to be a considerably more recent immigrant to this region, likely arriving in the Pleistocene, during the past 1–2 mya (Figures 1–2), well after the last major uplift of the Andes, which occurred until *circa* 3.0 mya (Gregory-Wodzicki 2000). Thus, it is likely that a dispersal event across the North Andes is responsible for the cis-Andean distribution of *Bassaricyon alleni*. This supports the idea that the Andes and the trans-Andean Neotropics (the western side of South America, and Central America) serve as continuous pumps of diversity into the Amazon, as proposed in other vertebrate groups such as tanagers and woodcreepers (e.g. Sedano and Burns 2010, Weir and Price 2011). The western boundary of the Amazon with the Andes and close proximity to the Chocó and Central America contribute to an influx of species from these regions into the Amazon and this influx seems to be a principal driver of the high diversity of the western Amazon and the eastern slopes of the Andes (Patterson et al. 2012).

One species of olingo, *Bassaricyon medius*, is distributed in the Chocó forests to the west of the Western Andes of Colombia and Ecuador, as well as in tropical forests of eastern Panama in Central America (Figure 12). For vertebrates, this is a common pattern: the Chocó has closer biogeographic affinities with Central America than with other areas of South America (Ron 2000). Mammalian examples of a Chocó + Central America distributional pattern include many medium-sized species in the region, including *Nasua narica*, *Procyon lotor*, *Coendou rothschildi*, *Tamandua mexicana*, *Caluromys derbianus*, and *Philander opossum* (Eisenberg 1989, Brown 2004, Voss 2011, Voss et al. 2013, Marín et al. 2012). That these various distributions result from multiple biogeographic events is evidenced by the dissimilar evolutionary divergence timings involved, but the GABI and Northern Andean uplift no doubt are key events that collaborated to generate these co-distributions. The divergence between the two subspecies of *Bassaricyon medius* is recent (*circa* 1.0 mya, Figure 1), but considering both subspecies are recorded in Colombia, it seems possible that *Bassaricyon medius* entered Panama quite recently, perhaps penetrating the North American continent as far as the distribution of the Mesoamerican endemic taxon *Bassaricyon gabbii*. The location of the geographic boundary between *Bassaricyon medius* and *Bassaricyon gabbii* in Panama is not yet clear, and the nature of interaction between these species, if any, at this boundary, will be a very interesting subject for further investigation.

The last species of olingo to consider, *Bassaricyon gabbii*, is a Mesoamerican endemic, distributed from Nicaragua to western Panama and recorded primarily in montane contexts: the Nicaraguan highlands, Costa Rican cordilleras, and Chiriqui Mountains. The eludication of the phylogenetic relationship, depth of divergence (we estimate a *circa* 2.0 mya divergence between *Bassaricyon gabbii* and the lowland species-pair *Bassaricyon medius*/*Bassaricyon alleni*) and the distinctive morphological features of *Bassaricyon gabbii* allow us to recognize it as the only carnivore species endemic to this region of Central America, although many vertebrate species, especially birds, reptiles and amphibians, and small mammals, are endemic to this same region (Savage 1966, 1982, Slud 1964, Stiles and Skutch 1989, Carleton and Musser 1995). As noted by Carleton and Musser (1995:357–358), “some have attributed the high endemism to the possible isolation of the Talamanca-Chiriquí region as an island, or a series of islands, within the Panamanian portal prior to complete closure and late-Pliocene formation of the landbridge” (citing McPherson 1985, 1986, among others). This vision of insular or archipelagic diversification in *Bassaricyon* during the GABI may provide insight into the early splits in the genus that ultimately gave rise to the principal modern lineages so far identified in the genus: *Bassaricyon neblina* in the Andes of northwestern South America, *Bassaricyon gabbii* in the Nicaragua-W Panama highlands, and *Bassaricyon medius*/*Bassaricyon alleni* in the Neotropical lowlands primarily in South America (southward from eastern Panama). Additional geographic surveys, specimen collecting, and specimen-based comparisons are needed to better understand the nature of differentiation in *Bassaricyon gabbii* across different Central American cordilleras, and the true easternmost extent of its distribution, where it may co-occur with or abut the range of *Bassaricyon medius*.

### Additional Olinguito study priorities

Our studies of Olinguito specimens in museums reveal a remarkable pattern of geographic variation, allowing for the delineation of four distinctive subspecific taxa distributed in separate biogeographic regions of the Andes of Colombia and Ecuador. Additional study is needed to more fully evaluate the level of genetic divergence between different Olinguito subspecies, especially for *Bassaricyon neblina ruber*, perhaps the most isolated and distinctive of the four (Figures 3, 9–10, 13–16).

**Figure 24.**
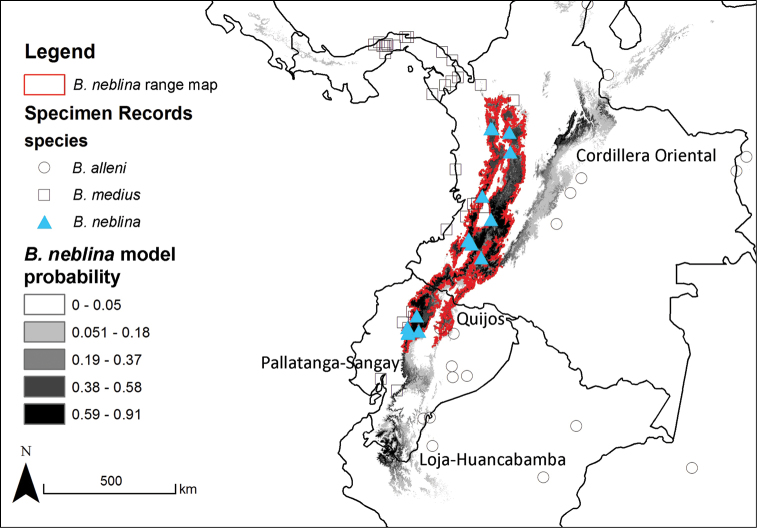
Selected priority areas to search for Olinguitos. Areas mentioned in the text with appropriate cloud forest habitats. A) Cordillera Oriental, the eastern branch of the Andes in Colombia. B) Quijos, a county on the eastern side of the Andes in Ecuador. C) Pallatanga-Sangay in central Ecuador. D) The Loja-Huancabamba region of the Andes in southern Ecuador and northern Peru.

Our bioclimatic analyses (Figures 11–12) also identify a number of high-priority candidate regions where further exploration is needed to assess whether additional populations of the Olinguito, or other distinctive high-elevation *Bassaricyon* populations, are present (Figure 24). One of these is the Colombian Eastern Andes, or Cordillera Oriental, the eastern branch of the Andes in Colombia. Olinguitos are recorded from the Western and Central Andes of Colombia, but not yet from the Eastern Andes, an area of substantial montane biotic endemism, where only *Bassaricyon alleni* is known to occur. Another survey priority is the Quijos region of Ecuador, a county and river situated on the eastern side of the Andes, which comprises relevant cloud forest habitats (Quijos is an old, pre-Spaniard name for the indigenous community in the area). This region deserves greater attention and contains the important Papallacta region discussed by Voss (2003). The Pallatanga-Sangay region in the Central Andes of Ecuador is another important priority study area; Pallatanga is an important mammal type locality (Tomes 1860), and Sangay is a national park with peculiar cloud forest mammal representation (Fonseca et al. 2003, Lee et al. 2011). Finally the Loja-Huancabamba, a low elevation region of the Andes in southern Ecuador and northern Peru has potential as Olinguito habitat. Though situated on the eastern side of the Andes, this region was recognized as biogeographically important by Chapman (1926). Patterson et al. (1992) showed little differentiation between *Artibeus* from the western slope of the Andes and the Marañón valley in this area of northern Peru, and Pinto (2009) inferred potential cases of east-west dispersal in vampire bats across both slopes of the Andes in this region of southern Ecuador, suggesting this could well be an area where the Olinguito could cross from the western to the eastern versant of the Andes.

Much remains to be learned about the Olinguito, including its distribution. The taxonomic description of this species is the first step toward further studies of its biology, and we look forward to future reports of additional discoveries from Andean cloud forests regarding this beautiful procyonid.

## Supplementary Material

XML Treatment for Bassaricyon neblina

XML Treatment for Bassaricyon neblina neblina

XML Treatment for Bassaricyon neblina osborni

XML Treatment for Bassaricyon neblina hershkovitzi

XML Treatment for Bassaricyon neblina ruber

XML Treatment for Bassaricyon gabbii

XML Treatment for Bassaricyon alleni

XML Treatment for Bassaricyon medius

## Appendix 1

### Morphometry

**Table A1.** PCA for male *Bassaricyon* skulls: variable contributions for the PCA axes that are graphically represented in Figure 6.

Variable	PC1	PC2
p2W	0.877	-0.077
p3W	0.768	0.004
p4W	0.600	-0.267
P4L	0.306	-0.412
P4W	0.526	-0.245
M1L	0.136	-0.427
M1W	0.336	-0.475
m1L	0.079	-0.241
m1W	-0.069	-0.750
m2L	-0.344	0.196
m2W	0.164	-0.330
CBL	0.733	0.317
ZYG	0.806	0.011
BBC	0.549	-0.050
HBC	0.458	-0.022
MTR	0.544	0.411
CC	0.912	-0.013
WPP	0.226	-0.464
LPP	0.704	-0.464
LAB	0.366	0.736
EAM	0.368	0.825
Eigenvalue	0.035	0.030
Variance (%)	30.1	25.8

**Table A2.** PCA for female *Bassaricyon* skulls: variable contributions for the PCA axes that are graphically represented in Figure 7.

Variable	PC1	PC2
p1W	-0.389	0.424
p2W	-0.765	0.341
p3W	-0.663	0.560
p4W	-0.783	0.068
P2W	-0.745	0.342
P3W	-0.724	0.221
P4L	-0.649	-0.314
P4W	-0.631	-0.370
M1L	-0.565	-0.275
M2L	-0.550	-0.003
M2W	-0.606	-0.324
m1L	-0.637	-0.200
m1W	-0.644	-0.524
m2L	-0.452	-0.166
m2W	-0.665	-0.302
CBL	-0.348	0.579
ZYG	-0.231	0.402
BBC	-0.317	0.210
MTR	-0.557	0.389
CC	-0.511	0.249
WPP	-0.342	-0.474
LPP	-0.170	-0.216
LAB	-0.018	0.733
EAM	0.066	0.743
Eigenvalue	0.034	0.021
Variance (%)	29.1	18.2

**Table A3.** DFA for male skulls of *Bassaricyon gabbii*, *Bassaricyon medius*, and *Bassaricyon alleni*, including correlation coefficients for the axes that are graphically represented in Figure 8. (Wilks’ Lambda: 0.11054 approx. F (16,58) = 7.2781 p < 0.0000).

	CV 1	CV 2	Wilks’ Lambda	Partial Lambda	F-remove (2,29)	p-value	Toler.	1-Toler. (R-sq)
p1W	0.249	-0.598	0.125	0.885	1.887	0.170	0.526	0.474
P3W	-0.644	-0.349	0.156	0.708	5.971	0.007	0.757	0.243
M2L	0.215	0.317	0.115	0.958	0.635	0.537	0.500	0.500
m1W	0.089	0.062	0.111	0.996	0.063	0.939	0.540	0.460
HBC	-0.855	0.207	0.181	0.610	9.267	0.001	0.653	0.347
ZYG	-0.274	0.174	0.119	0.933	1.048	0.363	0.916	0.084
WPP	0.289	-0.890	0.181	0.612	9.191	0.001	0.878	0.122
LPP	0.551	0.213	0.134	0.828	3.016	0.065	0.660	0.340
Eigenvalue	3.750	0.904						
Variance (%)	80.5	19.5						

**Table A4.** PCA for *Bassaricyon neblina* skulls: variable contributions for the PCA axes that are graphically represented in Figure 9.

Variable	PC1	PC2
CBL	-0.682	0.632
ZYG	-0.804	-0.185
BBC	-0.671	-0.373
HBC	-0.214	-0.689
MTR	-0.644	0.664
CC	-0.879	0.186
WPP	-0.194	-0.622
LPP	-0.854	0.461
LAB	-0.332	0.562
EAM	0.531	0.762
Eigenvalue	0.016	0.013
Variance (%)	39.7	33.0

## Appendix 2

**Table:** List of localities discussed in text and used in niche modeling analyses (visual records excluded from modeling).

taxon	latitude, longitude	locality
*Bassaricyon gabbii*	10.367, -83.850	Costa Rica: 9 mi NNW Roxana on Rio Chirripo
	9.800, -83.900	Costa Rica: Estrella de Cartago, 6 or 8 miles south of Cartago near the source of the Rio Estrella, altitude of ca. 4,500 feet.
	9.717, -84.267	Costa Rica: Fila Geronimo
	10.250, -84.433	Costa Rica: Lajas Villa Quesada
	8.783, -82.950	Costa Rica: Las Cruces Reserve (visual record only)
	10.100, -83.433	Costa Rica: Monteverde, Puntarenas Prov., T Guindez pasture
	10.467, -84.000	Costa Rica: Puerto Viejo, Rio Sarapiqui
	10.467, -84.017	Costa Rica: Rio Sarapiqui,Puerto Viejo, 300FT
	9.950, -84.050	Costa Rica: San Gerardo
	9.600, -82.783	Costa Rica: Talamanca (see Allen 1908)
	15.367, -88.733	Guatemala: La Sierra del Merendón (visual record only)
	14.983, -85.933	Honduras: Sierra de Agalta (visual record only)
	13.150, -86.150	Nicaragua: Jinotega, 16 km E, 5.5 km N Hacienda la Trampa
	12.917, -85.917	Nicaragua: Matagalba
	12.733, -85.733	Nicaragua: Rio Grande, south of Tuma, altitude below 1000 ft, Atlantic slope
	13.017, -86.000	Nicaragua: Tepeyac Matagalpa
	8.783, -82.417	Panama: Chiriqui, Boquete
	8.850, -82.567	Panama: Chiriqui, Cerro Punta, Casa Tilley
	8.850, -82.767	Panama: Chiriqui, Osta Clara
	8.883, -82.567	Panama: Chirirqui, between Rio Chiriqui Viejo and Rio Colorado, on a hill known locally as Cerro Pando, elevation 4800 feet, ca. 10 miles from El Volcan.
	9.300, -82.400	Panama: Almirante, Bocas del Toro
*Bassaricyon medius orinomus*	7.300, -75.383	Colombia: Puerta Valdivia
	7.833, -76.500	Colombia: Antioquia, Usaba, Villa Arteage, 130 m
	9.150, -79.850	Panama: Barro Colorado Island
	8.867, -79.717	Panama: Caimito
	8.950, -77.817	Panama: Campo Sasardi, 4 miles, W Mulatupo, Camarca de San Blas
	7.817, -77.717	Panama: Cana, in the mountains of the east
	9.283, -79.967	Panama: Canal Zone Rio Indio near Yatun
	9.167, -79.417	Panama: Cerro Azul, La Zumbadora
	9.200, -80.133	Panama: Colón Salud
	7.517, -78.167	Panama: Darien Jaque, near junction Rio Jaque and Rio Imamado
	7.950, -77.500	Panama: Darien Rio Paya (mouth)
	8.083, -77.283	Panama: Darien Tacarcuna, casita camp
	9.450, -79.067	Panama: Mandinga
	9.167, -79.667	Panama: Mouth of Rio Hondo, near Juan Mina
	7.817, -77.717	Panama: Mt Pirri, near head of Rio Limon
	9.217, -79.683	Panama: near Palenque
	9.150, -79.733	Panama: Parque National Soberania, lowland forest.
	9.450, -78.967	Panama: Rio Carti
	8.667, -77.467	Panama: San Blas Armila, Quebrada Venado
*Bassaricyon medius medius*	3.350, -77.000	Colombia: Agua Sucia, Rio Cajambre
	4.950, -77.367	Colombia: Chocó, Rio Baudo, Rio Saudo
	3.667, -76.400	Colombia: Dagua
	3.783, -76.767	Colombia: Jimenez, mountains inland of Chocó
	2.883, -77.667	Colombia: Rio Saija, La Boca
	3.800, -76.633	Colombia: Valle del Cauca, Sabaleta
	-0.433, -78.967	Ecuador: Cotopaxi, Las Pampas
	-2.167, -79.900	Ecuador: Guayas, Guayaquil
	-2.567, -79.350	Ecuador: Manta Real
	0.117, -78.833	Ecuador: Pichincha, near Gualea, W side Pichincha
	-0.050, -78.767	Ecuador: Pichincha, near Mindo
	-0.250, -79.150	Ecuador: Santo Domingo de los Tsachilas
*Bassaricyon alleni*	-13.633, -68.750	Bolivia: Alto Madidi
	-17.000, -64.000	Bolivia: Buena Vista
	-16.800, -64.950	Bolivia: Campamento Yuqui
	-11.567, -67.133	Bolivia: Cotoca Camp
	-17.100, -64.783	Bolivia: Sajta
	-11.400, -69.017	Bolivia: San Sebastien
	-17.450, -63.850	Bolivia: Santa Cruz, Ichilo, Rio Isamo, 400 m
	-4.150, -60.117	Brazil: Amazonas, Campina do Tupana (visual record only)
	-8.667, -72.783	Brazil: Acre
	-7.432, -73.661	Brazil: Acre, Serra do Divisor N.P.
	-5.083, -61.467	Brazil: Amazonas, Capina do Igapo Acu, 256km s of Manaus
	-5.200, -69.316	Brazil: Amazonas, Cujubim S.D.R., Curuena
	-7.467, -71.100	Brazil: Amazonas, Gregorio River E.R.
	-8.417, -72.400	Brazil: Amazonas, Igarape´ Porangaba
	-3.883, -60.617	Brazil: Amazonas, Modulo PPBio Manaquiri (visual record only)
	-5.450, -69.267	Brazil: Amazonas, Uacari S.D.R. (visual record only)
	4.667, -73.067	Colombia: Guaicaramo, on the Llanos of Villavicencio, east of Bogota
	3.100, -73.917	Colombia: Macarena Mountains
	10.583, -72.867	Colombia: Magdalena, Valledupar, Villanueva, Rio Cesar
	4.150, -73.450	Colombia: Medina
	10.583, -72.867	Colombia: Sierra Negra, Magdalena, Valledupar, Villanueva
	-3.517, -78.450	Ecaudor: Gualaquiza
	-3.483, -78.233	Ecuador/Peru: Coangos
	-0.633, -77.417	Ecuador: Avila
	-1.733, -77.483	Ecuador: Jatun Yacu, Rio Rutuno
	-0.450, -77.883	Ecuador: Napo, Baeza
	-2.067, -76.967	Ecuador: Pastaza, Rio Bobonaza, Montalvo
	-2.117, -77.450	Ecuador: Road Sarayacu, Copataz
	5.783, -57.633	Guyana: Bastrica (= Bartica) on the Essequibo River in British Guiana
	4.733, -58.850	Guyana: Burro-Burro River, Iwokrama Forest
	-10.667, -73.900	Peru Uvini, Rio Cosireni
	-8.833, -74.683	Peru: Aguas Calientes, etc.
	-10.133, -71.217	Peru: Balta
	-11.867, -72.783	Peru: Cashiriari
	-11.900, -71.367	Peru: Cocha Cashu
	-12.833, -69.300	Peru: Explorer’s Inn Reserve
	-11.050, -75.317	Peru: Junin, Chanchamayo, 1200 m
	-3.767, -73.250	Peru: Loreto, Iquitos, Santa Rita 120 m
	-5.517, -74.367	Peru: Loreto: Rio Ucayali, Yarinacocha
	-10.383, -75.450	Peru: Pasco, Cerros de Yanachagua
	-7.633, -75.017	Peru: Pisqui R, Ucalayi
	-10.067, -75.533	Peru: Pozuzo
	-11.783, -72.700	Peru: San Martin
	-13.150, -69.600	Peru: Tambopata
	-4.450, -78.150	Peru: Tseasim (Aguaruna village), Rio Huampami of Rio Cenepa
	4.900, -67.800	Venezuela: Munduapo, R. Orinoco, Venezuela
	2.250, -65.283	Venezuela: T. F. Amazonas, 108 km SE, Rio Mavaca,
	5.400, -67.650	Venezuela: T. F. Amazonas, 32 km SSE Puerto Ayacucho, Raya
	8.167, -72.167	Venezuela: Tachira 45 km N & 6 km E San Cristobal
*Bassaricyon neblina*	3.253, -76.160	Colombia, Los Alpes, Florida
	6.333, -76.117	Colombia: Antioquia, Urrao, Rio Ana
	6.333, -76.167	Colombia: Antioquia, Urrao, Rio Urrao
	5.545, -75.496	Colombia: Castillo Mts
	2.583, -76.917	Colombia: Cauca, Gallera
	2.533, -76.950	Colombia: Cauca, Munchique, 2000 m
	2.533, -76.883	Colombia: Cauca, Sabanetas
	4.049, -76.458	Colombia: El Duende Regional Reserve
	2.417, -76.817	Colombia: El Tambo
	1.950, -76.483	Colombia: Huila, San Agustin, San Antonio
	6.217, -75.533	Colombia: Santa Elena
	-0.617, -78.983	Ecuador: Cotopaxi, La Cantera in the trail to Sigchos
	-0.583, -78.983	Ecuador: Cotopaxi, near La Cantera in the trail to Sigchos
	-0.417, -79.000	Ecuador: Cotopaxi, Otonga
	-0.533, -78.650	Ecuador: S Domingo Trail, Las Maquinas
	-0.021, -78.684	Ecuador: Tandayapa
